# Self-Quenching Behavior of a Fluorescent Probe Incorporated within Lipid Membranes Explored Using Electrophoresis and Fluorescence Lifetime Imaging Microscopy

**DOI:** 10.1021/acs.jpcb.2c07652

**Published:** 2023-02-21

**Authors:** Sophie A. Meredith, Yuka Kusunoki, Simon D. Connell, Kenichi Morigaki, Stephen D. Evans, Peter G. Adams

**Affiliations:** † School of Physics and Astronomy, University of Leeds, Leeds LS2 9JT, U. K.; ‡ Astbury Centre for Structural Molecular Biology, University of Leeds, Leeds LS2 9JT, U. K.; § Graduate School of Agricultural Science and Biosignal Research Center, Kobe University, Rokkodaicho 1-1, Nada, Kobe 657-8501, Japan; || Graduate School of Agricultural Science, Kobe University, Rokkodaicho 1-1, Nada, Kobe 657-8501, Japan

## Abstract

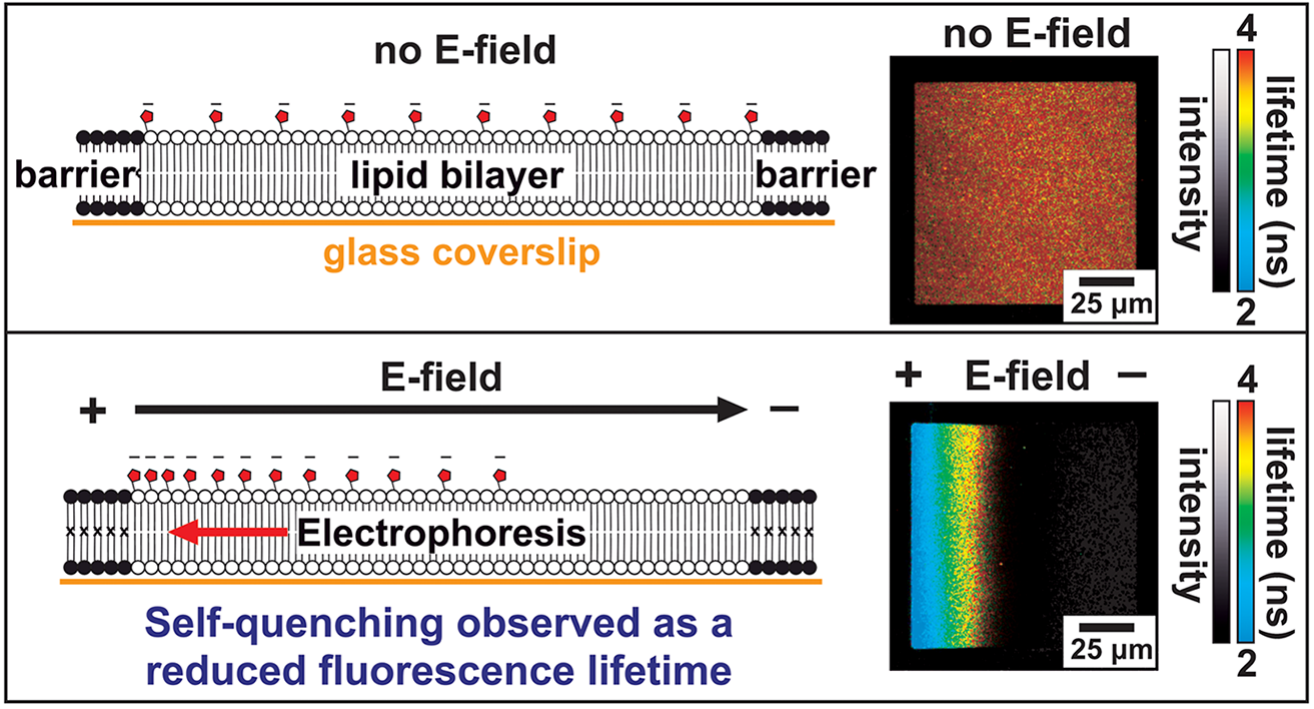

Fluorescent probes are useful in biophysics research to assess the spatial distribution, mobility, and interactions of biomolecules. However, fluorophores can undergo “self-quenching” of their fluorescence intensity at high concentrations. A greater understanding of concentration-quenching effects is important for avoiding artifacts in fluorescence images and relevant to energy transfer processes in photosynthesis. Here, we show that an electrophoresis technique can be used to control the migration of charged fluorophores associated with supported lipid bilayers (SLBs) and that quenching effects can be quantified with fluorescence lifetime imaging microscopy (FLIM). Confined SLBs containing controlled quantities of lipid-linked Texas Red (TR) fluorophores were generated within 100 × 100 μm corral regions on glass substrates. Application of an electric field in-plane with the lipid bilayer induced the migration of negatively charged TR-lipid molecules toward the positive electrode and created a lateral concentration gradient across each corral. The self-quenching of TR was directly observed in FLIM images as a correlation of high concentrations of fluorophores to reductions in their fluorescence lifetime. By varying the initial concentration of TR fluorophores incorporated into the SLBs from 0.3% to 0.8% (mol/mol), the maximum concentration of fluorophores reached during electrophoresis could be modulated from 2% up to 7% (mol/mol), leading to the reduction of fluorescence lifetime down to 30% and quenching of the fluorescence intensity down to 10% of their original levels. As part of this work, we demonstrated a method for converting fluorescence intensity profiles into molecular concentration profiles by correcting for quenching effects. The calculated concentration profiles have a good fit to an exponential growth function, suggesting that TR-lipids can diffuse freely even at high concentrations. Overall, these findings prove that electrophoresis is effective at producing microscale concentration gradients of a molecule-of-interest and that FLIM is an excellent approach to interrogate dynamic changes to molecular interactions via their photophysical state.

## Introduction

Fluorescent molecules (fluorophores) are commonly used as probes to report on the structure and interactions of biomolecules in many experimental systems. For example, fluorescently tagged lipids may be incorporated into natural biological membranes^1^ and artificial model membranes,^2−4^ where the fluorescence signal can provide information about the lipid mobility, the local physicochemical environment, and molecular concentrations.^5^ Given the ubiquity of fluorescence measurements in research, it is prudent to understand their accuracy and limitations and to take into consideration phenomena that may change the properties of fluorophores. One complication of fluorescent probes is that they can undergo “self-quenching”, i.e., reduction in emission intensity, as a result of interactions between each other.^6^ In fluorescence microscopy images, it is typical to assume that the concentration of the probe at each location is proportional to the intensity of the signal recorded, but it is known that this relationship breaks down at high local concentrations of the probe, where self-quenching effects become significant.^7−9^ In experimental investigations of biological systems, the fluorescence quenching effects are often mitigated by using a low concentration of fluorophore (typically 0.5%) and then assumed to be negligible; however, failure to account for this effect may lead to inaccurate estimates of fluorophore concentration and the misinterpretation of results.^10,11^ Another reason to study fluorescence quenching is because it is inherent to Förster resonance energy transfer (FRET) assays, a popular tool in biophysical research for measuring distances between two molecules.^12^ If unexpected quenching of the donor molecule occurred, then FRET assays would incorrectly underestimate the intermolecular distance.^13,14^ Finally, an understanding of the molecular basis for excitation energy dissipation is a critical aspect of photosynthesis research.^15,16^ Thus, a greater understanding of fluorescence quenching is of fundamental importance.

One challenge for assessing biophysical processes is to have an experimental model that allows control to be exerted over the distribution of biomolecules, and novel approaches are often required. In our case, for investigating the nature of self-quenching effects in biological systems, it would be useful to have a method to directly observe and manipulate the two-dimensional distribution of fluorescent molecules. Electric fields can be applied as a noninvasive method to perturb biological samples. Early studies in the 1970s demonstrated that an electric field applied across intact biological cells will induce the migration of charged molecules within natural biomembranes.^17,18^ Later, Sackman et al. showed that this idea could be applied to supported lipid bilayers (SLBs) as a way to manipulate this in vitro model of biomembranes.^19^ Boxer and co-workers extended the use of electrophoresis to SLBs that were deliberately confined in 2-D by constructing boundaries that limited diffusion, providing an even more controlled system.^20^ By applying an electric field parallel to the plane of an SLB, charged fluorophores migrate in the direction of the electrophoretic force and accumulate at one edge of a confined membrane. In a series of investigations, Boxer et al. demonstrated the generation of controllable concentration gradients of lipids,^20^ assessed the motion of membrane-tethered proteins,^7^ observed molecular demixing within membranes,^21,22^ and designed a geometrical “Brownian ratchet”.^23^ Other researchers showed that different molecular species could be laterally separated based on their different size and charge.^24−27^ Several groups showed that it is possible to control the distribution of fluorophores at equilibrium under the E-field by tuning experimental parameters such as the ionic strength of the aqueous buffer, the strength of the E-field, and the temperature.^20,25,28^ More recent work from Evans et al. has shown that both DC and AC fields and ratchets can be used to achieve over 20× increases in the concentration of lipids^8,29,30^ and membrane proteins.^30,31^ These previous studies used in-membrane electrophoresis in tandem with simple fluorescence microscopes to visualize the position of fluorophores, generally, for the purposes of studying the biophysics of lipids or membrane proteins. Fluorescence quenching effects were suggested as the reason that estimated concentration vs distance profiles observed during electrophoresis appeared to be an exponential growth function at low-to-moderate concentrations of fluorophores but “saturated” at high concentrations.^8,20^ For the study of lipid biophysics, further investigation of any quenching effects may not be required. However, for the study of the photophysics of fluorescent probes, it is fascinating that the electrophoresis method for creating controlled 2-D molecular distributions reveals a possible concentration-quenching effect. This approach appears to be more powerful and less laborious than the alternative of preparing a large sample set in which each sample contains a single defined concentration of fluorophores. If instruments which directly quantify fluorescence quenching could be used in tandem with a technique such as electrophoresis that generates a controlled 2-D distribution of fluorophores, then valuable details about the nature of the quenching versus concentration relationship could be revealed.

The “fluorescence lifetime” of a molecule is defined as the time for its excited state population to decay to a characteristic level (1/e) and is often represented by the Greek letter τ. Fluorescence lifetime is a useful property that can be measured to quantify the occurrence of photophysical processes such as energy transfer or quenching, as these processes cause changes to the decay rates of excited electronic states.^32^ Self-quenching is manifested as a reduction in fluorescence lifetime, in addition to the aforementioned decrease in fluorescence intensity, and can be accurately quantified as the ratiometric decrease in lifetime (τ/τ_0_). Importantly, fluorescence lifetime is independent of the molecular concentration (unlike fluorescence intensity measurements), so it can be highly accurate for assessing quenching effects. Fluorescence lifetime imaging microscopy (FLIM) is a specialized microscopy technique that acquires time-correlated single-photon counting data at every pixel of an image, allowing both fluorescence intensity and lifetime to be spatially correlated.^33,34^ Several studies have shown that FLIM can be a powerful technique to directly quantify the spatial distribution of quenched states, toward a greater understanding of quenching phenomena, FRET, and photosynthesis.^35−39^

For the current study, we hypothesized that in-membrane electrophoresis could be combined with FLIM as a powerful platform to set up concentration gradients of fluorescent molecules and directly quantify self-quenching effects, for the first time. We attempt to address the following questions: (i) Is it possible to directly observe self-quenching of fluorescent probes via changes to their fluorescence lifetime? (ii) Can we assess the onset of self-quenching effects over time as the system rearranges during electrophoresis? (iii) Can we convert fluorescence intensity profiles into concentration profiles with correction for quenching effects? (iv) Does the concentration of charged molecules deviate from an exponential growth function at high packing densities and can this provide hints about electrostatic and aggregation effects occurring during electrophoresis?

## Methods

### Preparation of Membrane Corrals

DiynePC and DOPC lipids were purchased as solids from Avanti Polar Lipids. The fluorescently tagged lipid TR-DHPE was purchased as a solid from Life Technologies. Template patterns were prepared as described in previous publications.^40,41^ Briefly, SLBs of DiynePC were formed on glass substrates by vesicle spreading, and then polymerization was conducted by UV irradiation through a photomask of the desired pattern, here, a 2-D array of 100 × 100 μm boxes. Lipid vesicles comprised of the specified ratio of TR-DHPE to DOPC lipids were generated with standard probe sonication procedures in pure water. To form membrane corrals, a suspension of lipid vesicles (at a concentration of ∼0.5 mg/mL total lipid) was incubated with a template pattern for 20 min and then rinsed with a low ionic strength buffer (purified water, adjusted to pH 7.5 using <0.1 mM HCl).

### In-Membrane Electrophoresis

**Figure 1:**
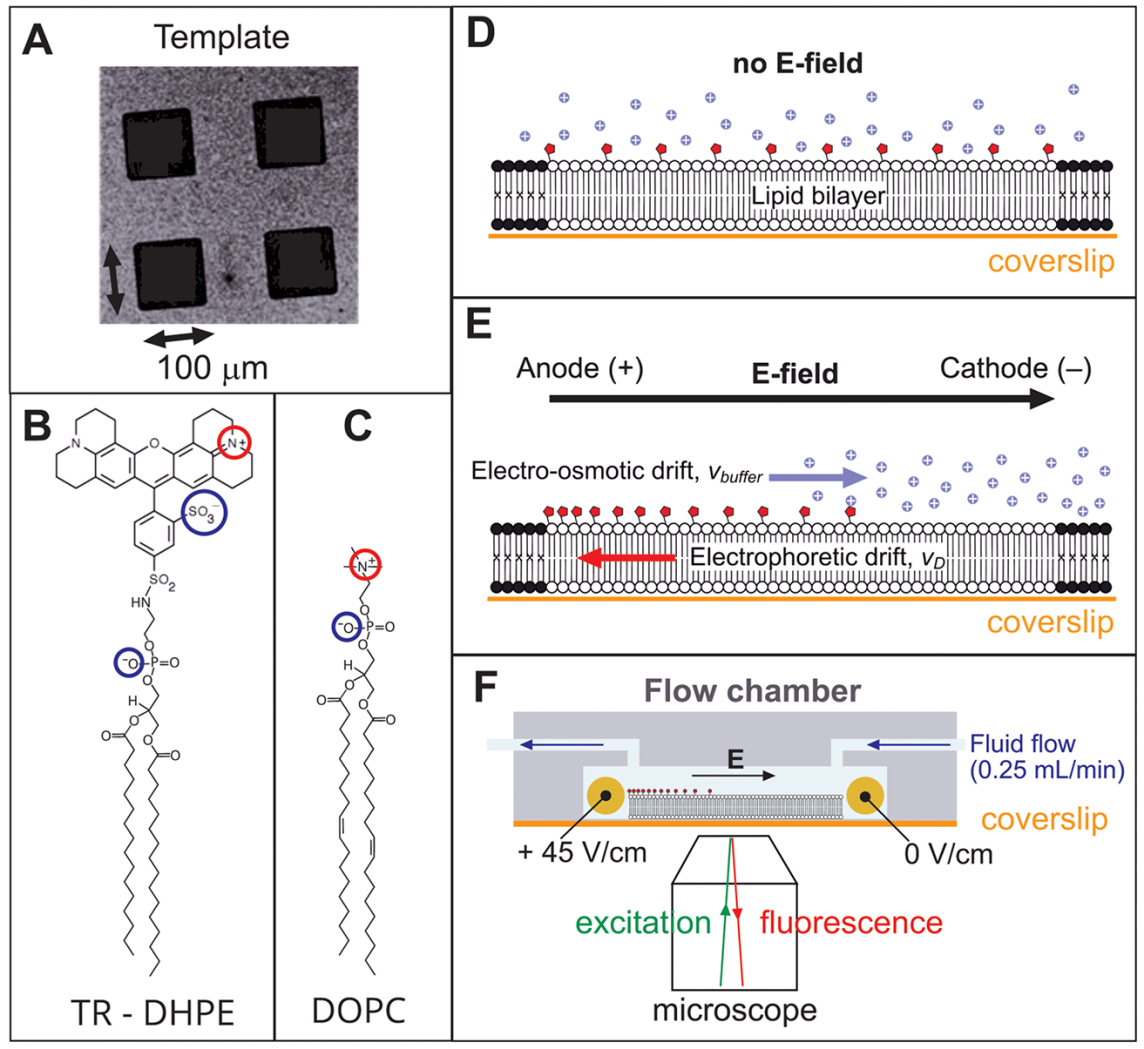
Concept for “in-membrane electrophoresis” experiments. (A) Example fluorescence microscopy image of the template pattern of photopolymerized DiynePC lipids (1,2-bis(10,12-tricosadiynoyl)-*sn*-glycero-3-phosphocholine), with excitation at 485 nm and collection of emission between 505 and 535 nm. These templates were generated in a microarray pattern by UV exposure through a photomask (see the Experimental Methods section for details). (B) Chemical structure of the fluorescent lipid TR-DHPE (Texas Red 1,2-dihexadecanoyl-*sn*-glycero-3-phosphoethanolamine). *Red circles* and *blue circles* mark positive and negative charges, respectively. (C) Chemical structure of the lipid DOPC (1,2-dioleoyl-*sn*-glycero-3-phosphocholine). (D) Schematic of a lipid bilayer confined by the barriers of the DiynePC template (black). In the absence of any electric field, TR fluorophores (red) will be uniformly distributed in this membrane corral, with a screen of ions (purple) close to the membrane surface. (E) As in (D) but with an applied E-field. (F) Schematic of the electrophoresis flow cell (not to scale), key parts labeled.

A custom-built electrophoresis chamber^8,31^ was used to hold a glass substrate under aqueous buffer in a suitable position for microscopy and allow the application of a controlled E-field (see Figure 1). The chamber was connected to a peristaltic pump via liquid outlets, and a continuous 0.25 mL/min flow of buffer was provided during electrophoresis experiments to prevent bubbling at the electrodes. Electrophoresis was performed on patterned membranes by applying an E-field (in-plane with the SLB) of 45 V/cm and monitored using a voltmeter throughout all experiments.

### Fluorescence Lifetime Imaging Microscopy (FLIM)

FLIM was performed using a Microtime 200 time-resolved fluorescence microscope (PicoQuant GmbH). This system used an Olympus IX73 inverted optical microscope as a sample holder with light passing into and exiting various filter units for laser scanning, emission detection, and timing electronics. A 561 nm excitation laser with a pulse width of 70 ps was driven in pulsed mode by a PDL 828 Sepia II burst generator module at a repetition rate of 10 MHz. A dichroic mirror and a bandpass emission filter with wavelength range 590–650 nm was used to define the emission channel. The detector was a hybrid Photomultiplier Tube, and the instrument response function was measured to have full-width at half-maximum of 100–120 ps. An excitation fluence of 0.012 mJ cm^–2^ was used for all measurements, which allowed sufficient fluorescence signal while limiting any singlet–singlet annihilation events (see Figures S1, S2, and S3). Images were acquired by scanning the laser using a galvanometric (FLIMbee) scanner and accumulating many frames of the same region (1 frame = 3.2 s). A standard FLIM image was 25 frames (80 s of exposure). We minimized the possibility of oxygen-dependent redox effects affecting the photophysical properties of the fluorophore by degassing all buffer solutions prior to use. Initial analysis of all FLIM data was performed with SymPhoTime software (PicoQuant). The mean amplitude-weighted lifetime of images or pixels, ⟨τ⟩, was calculated by generating fluorescence decay curves from accumulated photons and modeling the curve as a multiexponential decay function (excellent fits were achieved for all data, with chi-squared values < 1.1 and low residuals). Secondary graphical analysis was performed with OriginPro software. If concentration profiles were required (e.g., Figures 3-5), then images were exported from SymPhoTime software into two separate data channels: (i) a 2-D matrix of fluorescence intensity values at each x/y position and (ii) a 2-D matrix of fluorescence lifetime values at each x/y position, and then all y-values were averaged in OriginPro software (vertical averaging). These data were then plotted against the known distances and manipulated as described in the text.

## Results and Discussion

### Concept and Approach for Electrophoresis Using Micropatterned Supported Lipid Membranes

Photopolymerizable lipids were used to generate template patterns on glass coverslips that provide empty 100 × 100 μm square regions for lipid assembly (Figure 1A). The fluorescent probe used throughout this study was Texas Red (TR), attached to a lipid headgroup (DHPE) (Figure 1B), because this lipid-linked probe has an overall −1 charge, and previous studies have shown that it undergoes electrophoresis.^8,31^ The TR-DHPE was incorporated into vesicles mainly comprised of the net-neutral DOPC lipid (Figure 1C), typically at a 0.3–0.8% mole-to-mole ratio of TR-to-DOPC, and a solution of vesicles was incubated with the template to form patterned SLBs, termed “membrane corrals” (Figure 1D). Fluorophores within these corrals are expected to be highly mobile and homogeneously distributed throughout the membrane,^40,41^ and this was confirmed by fluorescence microscopy with photobleaching measurements (see Figure S1). When an electric field is applied parallel to an SLB, the negatively charged TR-DHPE lipids are expected to migrate toward the positive electrode and accumulate at the impenetrable edge of the membrane corrals (Figure 1E).^40−42^

Eventually, the system is expected to reach an equilibrium where the resulting concentration profile of fluorophores across the corral is the result of a balance of forces that work for (e.g., Lorentz force) and against (e.g., electroosmotic drag and random diffusion) the electric field. To maintain a consistent physicochemical environment during electrophoresis experiments, samples were maintained within a custom-built flow chamber under a constant flow of liquid (see schematic Figure 1F), and the strength of the E-field was set at 45 V/cm, as in previous studies.^8,31^ In initial experiments, the optimal image acquisition parameters were determined, including finding a suitable laser power and sample exposure time that were high enough to produce high signal-to-noise images but yet low enough so that fluorophores experienced minimal photobleaching (see Figure S2) and ensuring that singlet–singlet exciton annihilation was avoided (Figure S3). These preliminary experiments confirmed that the quality and reproducibility of the micropatterned lipid membranes was excellent and established the microscopy parameters required for high-quality fluorescence measurements.

### Experiments Assessing the Self-Quenching of Texas Red Using in-Membrane Electrophoresis

**Figure 2:**
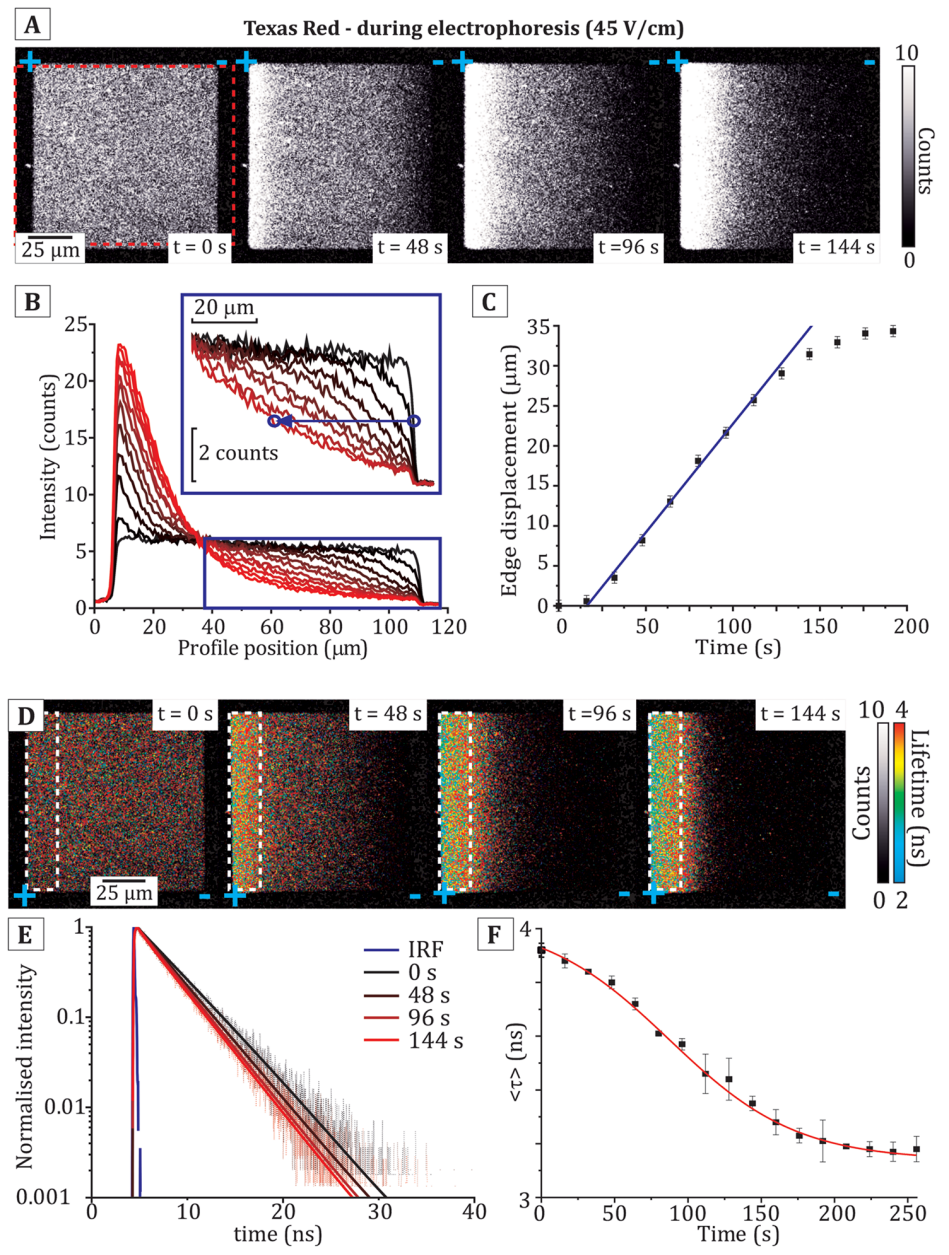
Kinetic analysis of the electrophoretic migration and fluorescence quenching of TR. (A) Time-lapse series of fluorescence intensity images of a lipid bilayer corral containing 0.28% (mol/mol) TR-DHPE after commencing the application of an electric field (45 V/cm). Each image represents a 5-frame acquisition (16 s exposure) at the standard excitation laser power (see Methods). (B) Average intensity profiles measured in the red, dashed box region in panel (A). Black-to-red lines represent increasing time points in a range from 0 to 192 s separated by 16-s intervals. Inset: The midpoint (half-maximum intensity) of the “moving edge” of fluorescence at the right side of the corral is measured for each time point (blue circle). (C) Graph showing the displacement of the moving edge measured in (B) with increasing time points. The drift velocity, *V*_drift_, is obtained from a linear fit (blue line). (D) Time-lapse series of FLIM images, as in (A) except including a color-scale for fluorescence lifetime. (E) Fluorescence decay curves obtained by accumulating photons collected at the edge of the bilayer (white dashed regions in (D)) after the application of the E-field, at the time points noted. (F) The fitted lifetime, ⟨τ⟩, at the edge of the corral as analyzed in (E), plotted against time after the application of the E-field.

To determine the effectiveness of our experimental system for directing the migration of lipids, first, we quantified the electrophoretic movement of TR in membrane corrals from the fluorescence intensity, and later, we assessed the self-quenching from the reduced fluorescence lifetimes. Figure 2A shows a series of fluorescence intensity images of a membrane corral containing 0.28% (mol/mol) TR taken at defined time points after the electric field was switched on. Each image was acquired with a short exposure time (16 s per image) to allow short time-steps for capturing changes which may occur quickly. Initially, the fluorescence intensity in the corral was homogeneously distributed within the square corral region (∼6 cts/pix). At later time points, the fluorescence intensity was observed to migrate toward the positive electrode, as expected for negative molecules, increasing up to a maximum of ∼23 cts/pix at the left edge of the corral while simultaneously decreasing to ∼1 cts/pix at the right edge of the corral after 192 s (Figure 2B). The electrophoretic drift velocity was calculated by tracking the displacement of a “moving edge” of fluorescence intensity as it travels toward the positive electrode, as detailed in previous studies.^43^

The displacement of the moving edge of TR was approximated by measuring the position of the half-maximum intensity in each frame (*inset*, Figure 2B). The relationship of fluorophore displacement versus time (Figure 2C) was found to be sigmoidal and may be explained by a three-stage process: (i) an initial lag phase after switching on the electric field where the fluorophores accelerate up to a terminal drift velocity (*t* = 0–16 s), (ii) a phase where the fluorophores drift toward the edge of the trap at a constant maximum velocity (*t* = 16–128 s), and (iii) a final phase where the velocity decreases toward zero and the displacement tends toward an equilibrium position as the electrophoretic drift becomes balanced by the random diffusion of fluorophores (*t* = 128–192 s). The terminal drift velocity can be determined in phase 2, where the electrophoretic force is balanced by opposing forces (electroosmotic drag force due to counterion flow and frictional drag force due to lipids moving through an existing bilayer). The terminal drift velocity of TR was found by fitting a straight line to the roughly linear region (*blue line*, Figure 2C) as 0.28 ± 0.01 μm/s (fitted value ± uncertainty). To estimate the contribution of electroosmotic drag, the electrophoretic mobility, *μ*_EP_ = *V*_drift_*/qeE*, was calculated and compared to the mobility calculated from FRAP measurements, μ_FRAP_. The ratio of the two mobilities, α = μ_EP_/μ_FRAP_, was calculated as 0.70 ± 0.04 suggesting that combined opposing forces result in a 30% reduction in TR mobility. These values for the drift velocity and mobility of TR are similar to previous reports using in-membrane electrophoresis.^8^ Overall, this kinetic analysis of the migration of TR shows that our electrophoresis protocol was effective at inducing changes to the fluorophore concentration and similar to other published works.^8,20,30^

As the TR fluorophore becomes increasingly concentrated, it will eventually start to quench. A cursory examination of the intensity curve at the final time point (Figure 2B) does not seem to indicate any anomalous reduction or drop-off in intensity at the far left edge. To investigate this further, the fluorescence lifetime signal was investigated as it is sensitive to the photophysical state, i.e., the presence of quenching. Figure 2D overlays the intensity images with false color-scale to represent the fluorescence lifetime. At *t* = 0 s, the majority of pixels in the FLIM image are a *red color* representing a long fluorescence lifetime of ∼4 ns. At increasing time points, as the TR fluorophores migrate toward the positive electrode, a significant decrease in the fluorescence lifetime was evident as a subtle color shift from *red/green* to *green*/*blue* at the left edge of the corral. This visualization is good qualitative evidence for changes in the excited state lifetime of TR and self-quenching of its fluorescence at increased concentrations. To quantify the lifetime changes more robustly, photons were accumulated from a region-of-interest (ROI) at the high-concentration end of the trap (*white dashed* regions in Figure 2D), and histograms were plotted of the fluorescence decay at each time point (Figure 2E). An exponential function was fit to each decay curve to determine the average fluorescence lifetime, τ, from *F*(*t*) = ∑*A*_*i*_*e*^–*t*/*τ*_*i*_^ (mono- and biexponentials typically achieved the best fit). At increasing time points, the fluorescence decay curves became increasingly steep (*black* to *red* curves, Figure 2E), and the mean lifetime decreased from τ = 3.92 ns at *t* = 0 s to τ = 3.18 ns at 256 s. The onset of quenching was analyzed by plotting the fluorescence lifetime against time and was found to follow a decreasing sigmoidal trend (Figure 2F), with a good inverse correlation to the kinetic data for fluorescence intensity (increasing sigmoid in Figure 2C). This reduction in the fluorescence lifetime is correlated with the increase in fluorescence intensity at the left edge of the corral at later time points (Figure 2B) and is direct evidence that TR molecules interact with each other at sufficiently high concentrations leading to the nonradiative dissipation of energy. The shallow gradient of intensity and lifetimes across the corral suggests a gradual increase in quenching as molecular concentration increases, rather than a binary on/off state. Note that the absolute values calculated for lifetime in this analysis should not be considered exact because there is a moderate amount of noise in these images, due to the short acquisition times required for generating a large series of images. Later experiments use longer image acquisition times and accumulate data from multiple corrals to give higher confidence in lifetime values.

### Correcting for Self-Quenching by Converting Fluorescence Intensity Profiles to Concentration Profiles

**Figure 3:**
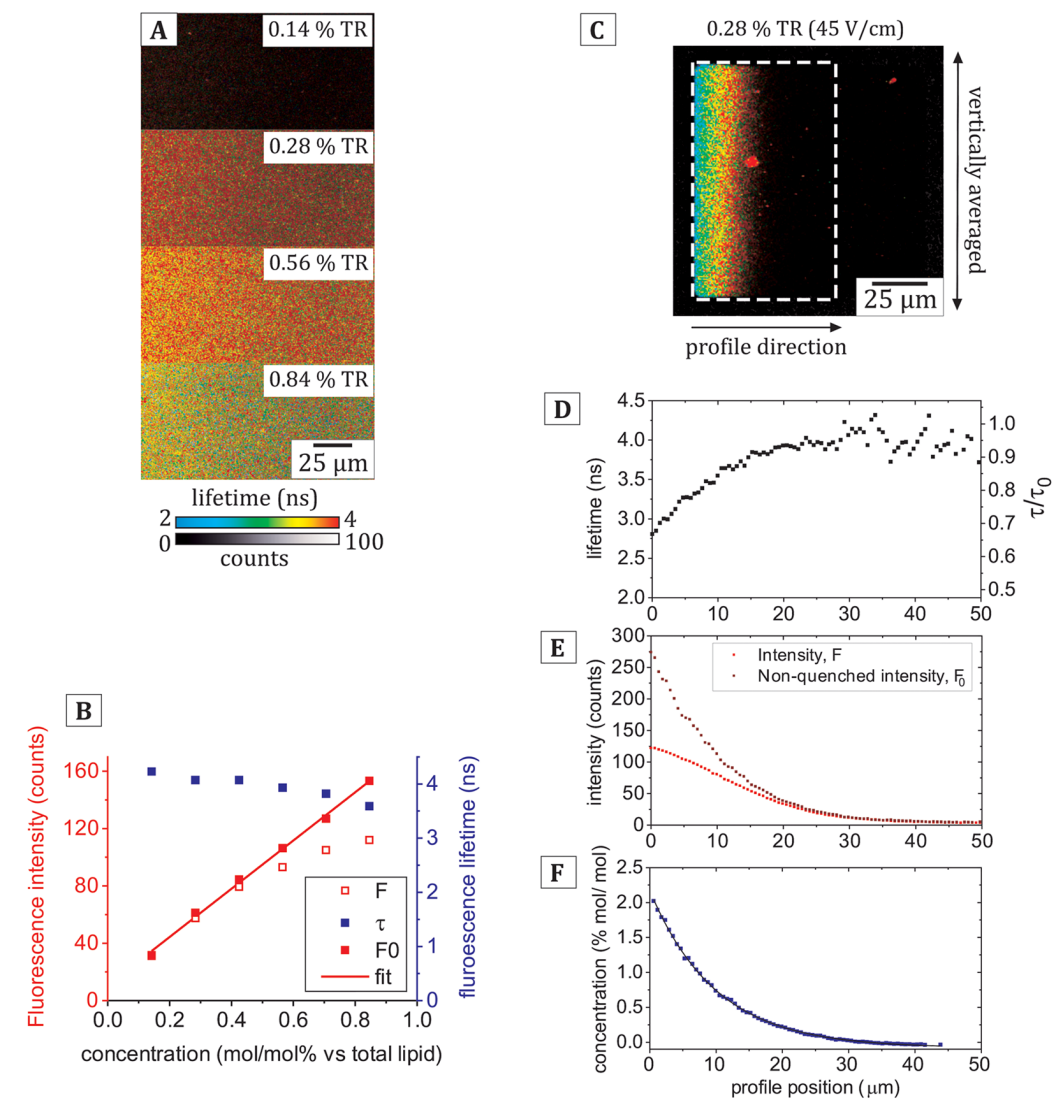
Demonstration of the method for generating concentration profiles from a FLIM image. (A) Example FLIM images of a sample series of SLBs containing TR at defined concentrations, as labeled (each image: 25 frames at standard laser power). (B) Graph showing the raw fluorescence intensity (open red squares) and the fluorescence lifetime (blue squares), as calculated from the average of all pixels in an image. The corrected fluorescence intensity, *F*_0_ (filled red squares), was calculated using eq 1 as described in the text, and the tabulated results are shown in Table S2 in the Supporting Information. The solid red line is a linear fit, *F*_0_ = *mC* + *Y*_0_, with the solution *m* = 168.3 and *Y*_0_ = 10.7. The fact that *Y*_0_ is not equal to zero is likely to be due to a low amount of fluorescence background. (C) Example FLIM image of a 0.28% (mol/mol) TR corral at equilibrium in an E-field. The white dashed box denotes the ROI from which horizontal profiles of lifetime and intensity were obtained by averaging the pixels accumulated vertically (typically 150 pix/vertical, improving the signal-to-noise). (D) Profile of fluorescence lifetime against the x-position obtained from the white dashed region in (C). (E) Profile of the raw fluorescence intensity profile (light red), *F*, obtained from the white dashed region in (C). The nonquenched intensity, *F*_0_ (dark red), was calculated from the profile for *F*(*x*) and the profile for τ(*x*) using eq 1 in the following form: *F*_0_(*x*) = *F*(*x*)·*e*^2 ln[τ_0_/τ(*x*)]^. (F) The concentration profile (blue) calculated from the data for *F*_0_ from (E) using the direct proportionality relationship between the molar concentration and nonquenched intensity of a fluorophore, *C* = (*F*_0_ – 10.7)/168.3. The solid black line is a fit to the monoexponential function: *C*(*x*) = *a*·*e*^–*bx*^ + *y*_0_ (*a*, *b*, and *y*_0_ are fitting constants).

After the observation that quenching of TR occurred during electrophoresis, we wished to quantify the concentration-quenching relationship. To do this, a more accurate representation of the relative concentration of the TR fluorophore within the lipid bilayer must first be obtained. So, a methodology was devised to convert the fluorescence intensity measured in each pixel of a FLIM image to a molecular concentration. All FLIM images reported later in this study were captured with a longer exposure time (80 s per image) than for the time-lapse image series in order to increase the measured number of counts and precision of subsequent analyses of the “steady state” of membranes (when the TR distribution was stable, before or after electrophoresis). As a first step, standard samples containing known concentrations of the fluorophore were needed to establish the concentration relationship. Therefore, a series of lipid membranes containing defined TR-DHPE concentrations, in a range from 0.14 to 0.85 mol/mol % of TR relative to DOPC, were formed on hydrophilic glass (without any template pattern). These SLBs containing relatively low concentrations of TR acted as simple control samples due to their high reproducibility and ease-of-use. It is not usually possible to prepare high-quality SLBs from lipid vesicles containing concentrations of TR > 1.5% due to electrostatic repulsive effects which cause defects in supported membranes,^44^ hence the usefulness of techniques like electrophoresis to generate higher concentrations. FLIM images of the SLBs containing 0.14–0.85% TR were acquired using the same settings as described for electrophoresis measurements (Figure 3A). The average fluorescence intensity and average lifetime were calculated for each image and the results were plotted as a function of concentration for the sample series (Figure 3B). In the absence of quenching, the fluorescence intensity is expected to increase linearly with concentration. However, this was not observed in the raw data because the fluorescence intensity was somewhat reduced as a result of quenching. The raw fluorescence intensity increased with TR concentration roughly linearly below ∼0.5% mol/mol, but the trend clearly starts to deviate above this (*open red square*s, Figure 3B). The fluorescence lifetime decreased gradually with increasing TR concentration (*blue square*s, Figure 3B). Established theory suggests that it should be possible to correct the raw fluorescence intensity (*F*) for quenching effects as observed from the reduction in fluorescence lifetime (τ), as follows (derivation provided in section 4 of the Supporting Information)
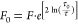
1where *F*_0_ is the calculated intensity for a nonquenched system, and τ_0_ is the lifetime estimated for nonquenched TR (τ_0_ = 4.2 ns, from measurement at very low TR concentration). The values calculated for *F*_0_ were replotted on the same graph (*filled red square*s, Figure 3B) and, in this case, the expected linear relationship with TR concentration was apparent.

This corrected fluorescence intensity was fit successfully to a straight line, *F*_0_*= mC + Y*_0_, where *m* is the fitted gradient, *C* is the concentration, and *Y*_0_ is the *y*-intercept. The gradient of the linear fit provides a conversion factor that allows *F*_0_ to be converted to *C* of Texas Red, for any FLIM images using the same acquisition settings. Using this relationship, the TR concentration could be calculated at each location in membrane corrals in FLIM images using the fluorescence intensity and fluorescence lifetime.

Next, this conversion was applied to membrane corrals undergoing electrophoresis, described below. Figure 3C shows a FLIM image of a membrane corral at electrophoretic equilibrium which had a starting concentration of 0.28% (mol/mol) TR, showing the direction of the profiles generated from left-to-right across the membrane (*white dashed region*). The mean values for fluorescence intensity and lifetime were calculated for each horizontal (x) position across the membrane corral by averaging vertical columns (y) of pixels. The resulting profiles for fluorescence lifetime and raw fluorescence intensity are shown in Figure 3D-E (*black* and *bright red* data points, respectively). The nonquenched fluorescence intensity (*F*_0_) profile was calculated using eq 1, as described above (*dark red data points* in Figure 3E). The mole-to-mole TR fluorophore concentration at each horizontal position (*blue data points* in Figure 3F) was then calculated from *F*_0_, as above. We found that the TR concentration in this example increased roughly exponentially from zero on the right side of the corral up to ∼2.5% (mol/mol) at the left side of the corral. This was correlated to a decrease in the fluorescence lifetime of TR from ∼4 ns to ∼2.5 ns from right-to-left.

### Consistency of Concentration Profiles Across Multiple Membrane Corrals

**Figure 4:**
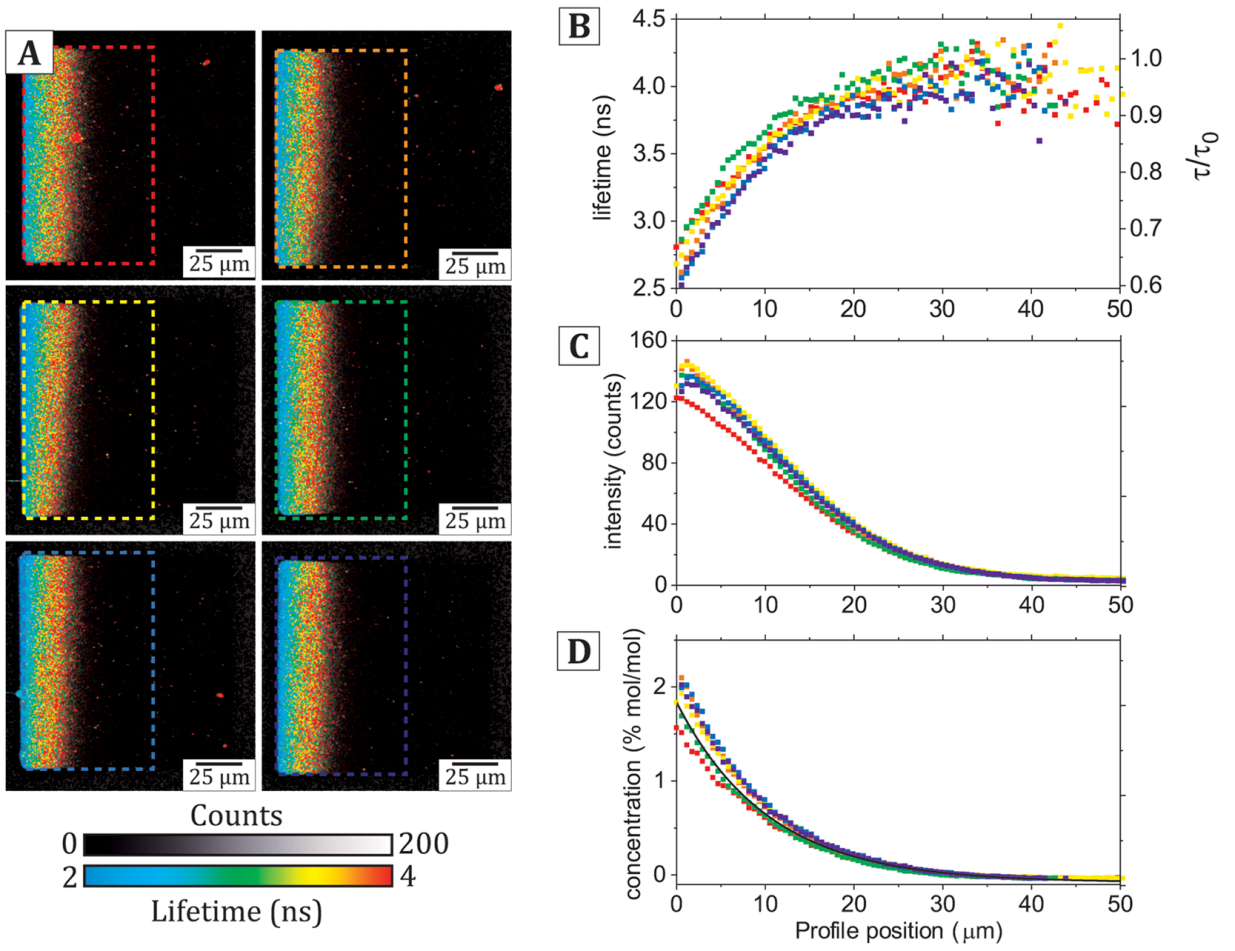
Comparison of the consistency of fluorescence lifetime profiles and calculated concentration profiles for multiple corrals within one sample. (A) FLIM images of six different corrals at equilibrium during electrophoresis (45 V/cm) (each image: 25 frames at standard laser power). This sample had a starting concentration of 0.28% mol/mol TR-DHPE to DOPC. The colored dashed boxes show the regions-of-interest from which lifetime and concentration profiles were obtained (as described in Figure 3). Different colored dashed boxes correspond to the different colored scatter plots in subsequent panels. (B) Multiple profiles of fluorescence lifetime vs x-position across the membrane corral, corresponding to the ROIs indicated in (A). (C) Multiple profiles of raw fluorescence intensity vs x-position. (D) Multiple profiles of the calculated concentration profiles, generated as described in Figure 3. The black line shows a fit of the combined dataset to a monoexponential function: *C*(*x*) = *a*·*e*^–*bx*^ (*a* and *b* are fitting constants).

To assess the variability of membrane behavior in response to electrophoresis, FLIM analysis was performed on multiple membrane corrals (*N* = 6) within one sample, as shown in Figure 4A. Fluorescence lifetime profiles and concentration profiles were calculated using the process described in the previous section (Figure 4B-D). Overall, the lifetime and concentration profiles were highly consistent across multiple corrals, as shown by the relatively narrow range of lifetimes/concentrations found at each particular position along the horizontal. For example, at *x* = 0, the lifetime ranges from 2.5 to 2.8 ns and concentration ranges from 1.5 to 2.0% TR (mol/mol). These results highlight the consistency of in-membrane electrophoresis as a method to control fluorophore concentration, showing that fluorophores accumulate in a predictable manner in response to the electric field for a single starting fluorophore concentration.

### Increasing the Initial Fluorophore Concentration Increases the Amount of Fluorescence Quenching Achieved during Electrophoresis

**Figure 5:**
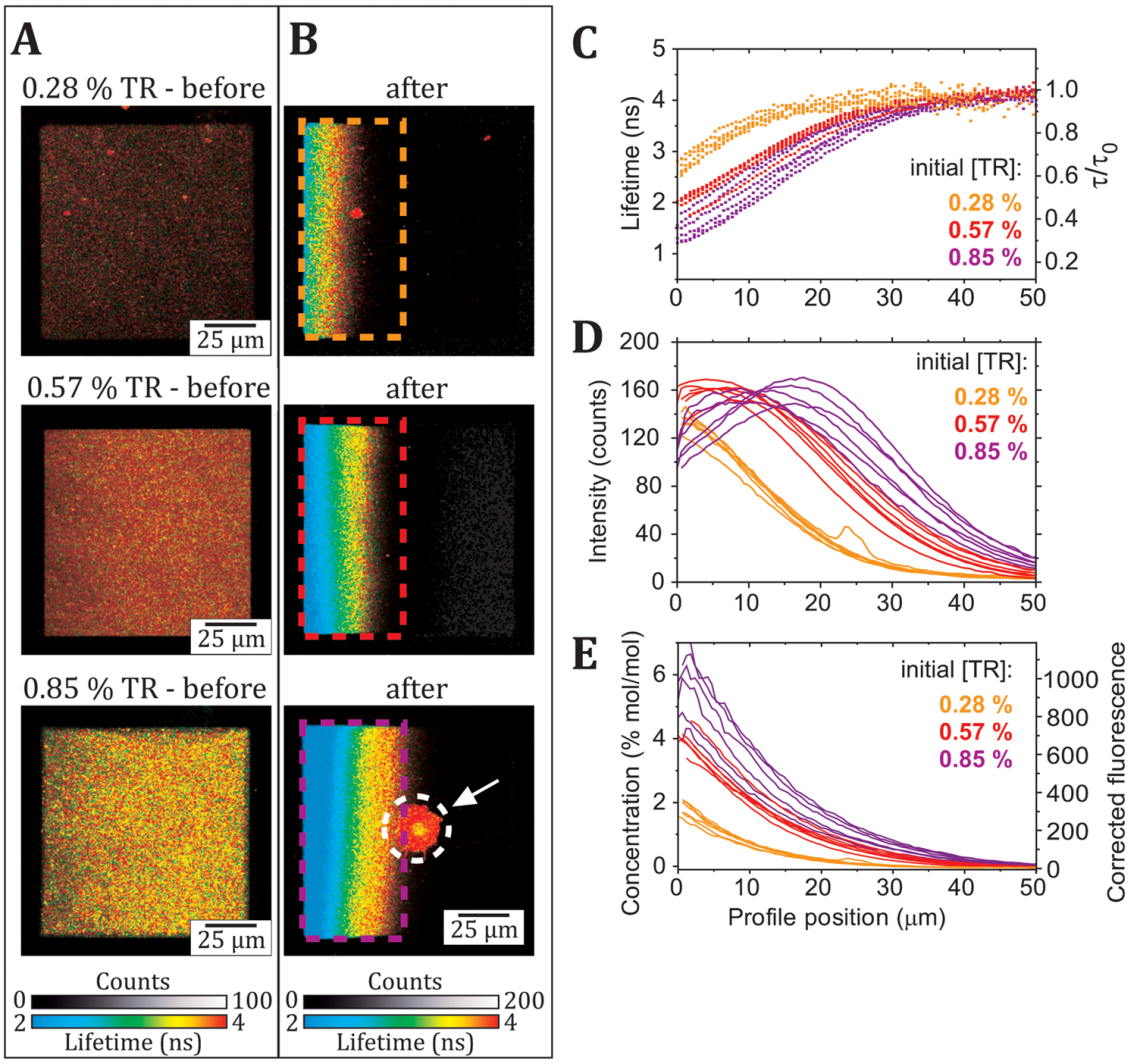
Comparison of the fluorescence properties of membrane corrals before and after electrophoresis for SLBs containing a range of starting concentrations of TR. (A) FLIM images of corrals containing either 0.28, 0.57, or 0.85 mol/mol % TR (relative to DOPC) before application of an E-field. (B) FLIM images of the corrals from (A) at equilibrium during electrophoresis (45 V/cm). The white arrow indicates a defect in the SLB that appeared during electrophoresis, and the related area (circled in white) was excluded from later analyses. (C) Multiple profiles of fluorescence lifetime vs x-position across the membrane corral, corresponding to the ROIs indicated in (B). The mean lifetime was used for this analysis, and for completeness, the multiexponential character of the fluorescence decay curves was analyzed elsewhere (see Figure S5). (D) Multiple profiles of raw fluorescence intensity vs x-position. (E) Multiple profiles of the corrected fluorescence intensity (right axis) and the equivalent calculated concentration (left axis), generated as described in Figure 3.

At this stage, we wished to test the limits of the membrane electrophoresis technique and asked the following questions: (i) What is the highest fluorophore concentration that can be achieved? (ii) Do we reach even greater levels of quenching due to these higher molecular concentrations? It is logical that a higher starting concentration of fluorescent probes would result in greater final fluorophore concentrations accumulated during electrophoresis, unless repulsive forces or molecular aggregation effects prevent this. Therefore, electrophoresis and FLIM analysis were performed for a series of membrane samples prepared using different starting concentrations of TR. FLIM images from membrane corrals containing 0.28%, 0.57%, and 0.85% TR (mol/mol relative to DOPC) before electrophoresis show that the fluorescence intensity increased and the fluorescence lifetime reduced with the increasing initial TR concentration (Figure 5A). From inspection of FLIM images of the same membrane corrals at electrophoretic equilibrium (Figure 5B), qualitatively, it was clear that increasing the initial TR concentration leads to an increased intensity and a wider band of fluorescence accumulating at the left edge of the membrane.

Correlated to these fluorescence intensity gradients, each corral had a characteristic *blue*-*green*-*yellow*-*red* color gradient representing the fluorescence lifetime change from left-to-right across the membrane with a much wider expanse of low-lifetime signal (*blue* pixels) for the higher initial concentrations. Some disruption to the membrane structure (*white arrow*, Figure 5B) occurred after electrophoresis only for the highest TR concentration sample (0.85%) and is likely to be the curvature-induced formation of lipid tubules (discussed in the Supporting Information, see Figure S4). Regions containing such defects were digitally excluded from further analyses of fluorescence data to allow fair comparisons between membranes. FLIM images of multiple membrane corrals were analyzed for each sample to assess the consistency, and the data are overlaid onto graphs comparing the profiles of raw fluorescence intensity and the fluorescence lifetime for different starting concentrations of TR (Figure 5C–D). The profiles of the raw fluorescence intensity (Figure 5D) show that the maximum intensity reached during electrophoresis was ∼120 counts/pix for a starting concentration of 0.28% TR, compared to ∼160 counts/pix for starting concentrations 0.57% and 0.85% TR. The data for the highest starting TR concentration of 0.85% was particularly revealing (*purple curve*, Figure 5D): following the intensity profile from right-to-left across the corral, one finds that the intensity increased rapidly from ∼20 counts/pix at an x-position of 50 μm to a maximum intensity of ∼160 counts/pix at *x* = 20 μm before decreasing to ∼110 counts/pix at the left edge of the corral, at *x* = 0. This decline in the fluorescence intensity at the left-hand edge of the corral, where the molecular concentration of the fluorophore is expected to be greater, indicates that at extremely high concentrations of TR the self-quenching becomes so significant that each additional fluorophore has a negative contribution to the measured intensity. The fluorescence lifetime profiles (Figure 5C) support this interpretation: all samples display fluorescence lifetimes of ∼4 ns on the right-hand side of the corral (i.e., *x* > 40 μm), but the lifetime is reduced to much lower values at the left-hand edge of the corral (at *x* = 0) for samples containing greater TR, to ∼2.7 ns, ∼2.0 ns, and ∼1.3 ns. This equates to a maximal lifetime reduction down to 30% of its original value (τ/τ_0_) for samples with the high initial TR concentrations of 0.85% (mol/mol). A deeper analysis of the multiexponential character of fluorescence decay curves extracted from the FLIM data suggests a gradual change in the quenching pathways due to increasing TR-TR interactions (Figure S5). The corrected fluorescence intensity and concentration profiles were then calculated from the raw intensity and lifetime data (Figure 5E) following the method described in the previous section. For the highest initial TR concentrations of 0.85% (mol/mol), the corrected fluorescence intensities were up to 10× the original measured fluorescence intensities, at ∼1000 counts/pix (Figure 5E, *right axis*), equating to quenching of the fluorescence intensity down to 10% of its original value (*F*/*F*_0_). This finding is in good agreement with the electrophoresis study of Bao et al.^8^ who observed quenching down to ∼13% of the fluorescence intensity for an ∼6% TR (mol/mol) concentration, as estimated with an empirical model of self-quenching. As expected, membrane corrals with a higher initial TR concentration resulted in a higher maximum concentration achieved during electrophoresis, and notably, the relative increase in fluorophore concentration was approximately consistent: for initial TR concentrations of 0.28, 0.57, and 0.85% (mol/mol) the maximum concentration reached during electrophoresis was ∼2.0, ∼4.0, and ∼6.0% (Figure 5E, *left axis*), respectively, an increase of approximately 7-fold in all three cases. This demonstrates the consistency of in-membrane electrophoresis as a method to increase fluorophore concentration and shows that changing the initial membrane concentration allows control over the final state of the membrane. Overall, these results show that the degree of quenching achieved with in-membrane electrophoresis can be controlled by simple modifications to the starting composition of the membrane. This could allow this platform to be used to investigate quenching over a wide dynamic range of concentrations for many other electrostatically charged fluorophores, such as different organic molecules or even membrane proteins, such as the light-harvesting pigment-protein complexes involved in photosynthesis in plants and bacteria.^38,39,45,46^

The limitations of the electrophoresis technique and the analysis methodology should be briefly discussed in a wider context. First, the electrophoresis technique has the requirement that the fluorescent probe must respond to an electric field; therefore, alternative lipid-linked molecules that have either (single or multiple) negative or positive charges are likely to be effective, but neutral molecules will not undergo electrophoresis. Bulky molecules that protrude above the membrane, such as tethered proteins,^7,27^ may be driven to migrate by the field-induced flow of buffer molecules, a process termed “electro-osmosis”. Second, the ability of a molecule to migrate within an SLB will depend upon the composition of the lipid membrane. More complex lipid mixtures, e.g., saturated lipids/cholesterol/charged lipids, could be investigated in future studies, and different levels of migration may occur depending on the membrane’s fluidity and phase structure.^21,22^ Third, we must acknowledge that FLIM data contains a moderate amount of noise and that this can lead to variations in the lifetime observed in the raw data, e.g., variations between the concentration profiles of samples expected to contain similar quantities of TR (Figure 5E). In our judgment, the best way to produce accurate results was to accumulate data from multiple samples and multiple corrals. To assess our noise and accuracy, one may observe the scatter plot of lifetimes considering many corrals overlaid, shown in Figure 5C. Here, the lifetime tends toward *y* = 4.1 ± 0.3 ns at *x* = 50 μm which equates to τ/τ_0_ of 0.98 ± 0.07 (where ± represents the peripheral data points). This is in very good agreement with the situation of zero quenching expected for very low TR concentrations (τ ≡ 4.2 ns and τ/τ_0_ ≡ 1). Thus, the precision of an individual measurement may vary with the noise experienced in the data, but the overall accuracy is high.

**Figure 6:**
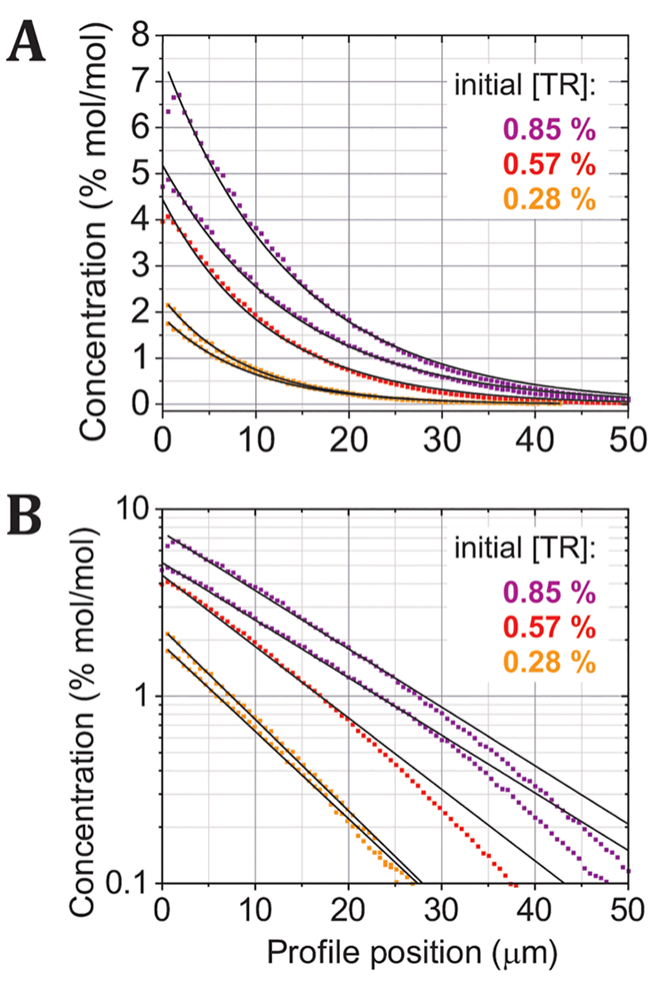
Assessing whether concentration profiles follow a monoexponential function. (A) Selected concentration profiles as a scatter plot, colored as in Figure 5. The black lines are a fit to a monoexponential function: *C*(*x*) = *a*·*e*^–*bx*^ (*a* and *b* are fitting constants), performed for each profile. (B) The same data and fitting as in (A), except displayed as a semilogarithmic plot.

One final point of interest is to consider how the interactions between charged particles within a lipid bilayer may change at very high concentrations. As noted earlier, at equilibrium during electrophoresis, we may expect the concentration profile of fluorophores to follow a monoexponential growth function. In a previous study using an initial 1% (mol/mol) concentration of TR-DHPE in similar lipid bilayers to our work, the authors noted that the quenching of the TR fluorescence intensity led to its deviation from an exponential profile during electrophoresis-induced accumulation.^20^ Whereas, they found that alternative fluorophores such as NBD-DHPE did not appear to quench during electrophoresis when using the same initial fluorophore concentration.^7,21^ So, the inherent photophysics of the specific fluorophore appears to play a major role. Previous studies suggested that both photophysical interactions, i.e., quenching, and also physicochemical interactions such as lipid aggregation could be the cause of the “rolling off” of the raw fluorescence intensity profiles as the fluorophores accumulate at the edge of a barrier.^7−9,20,21^ For the first time, we can attempt to decompose these two effects because of our direct calculation of the effective TR concentration, by correcting for quenching effects. A cursory look at the exponential fits of our data certainly suggests that this mathematical function is a good approximation when starting with the relatively low 0.28% starting concentration of TR (e.g., *black lines* in Figure 3F and Figure 4D). To take a deeper look, we can assess the quality of fit for multiple concentration profiles displayed both with standard linear axes (Figure 6A) and with semilogarithmic axes (Figure 6B). A semilog plot is useful because it allows clear visualization of exponentiality, as a straight line represents a monoexponential fit; whereas, a linear plot is more likely to reveal subtle deviations from a fit (as slight differences are less evident on a log-scale). The fit appears to be very good over a large portion of the graph (*x* ≈ 2 to 25 μm); however, there is a deviation from the fit at the very start and toward the end of the profiles. We also note that this deviation is most significant for membranes with the highest starting concentration of TR (*purple* plots). At the start of the profile, between *x* = 0 to 2 μm, there is a subtle deviation from the fit where the first one to two data points reveal a very slightly lower concentration than the exponential profile (shown most clearly in Figure 6A). This difference could suggest that electrostatic repulsion between the negatively charged TR is limiting greater accumulation at the very highest concentrations achieved. The fact that this deviation is only very subtle suggests that large aggregates of lipids do not occur. Between profile positions of 25 to 50 μm, there also appears to be a slight deviation away from a monoexponential (shown clearly in Figure 6B).

This is somewhat surprising, and we may only speculate on the explanation without further evidence. One possibility is that a biexponential reflects the fact that there are known to be two different chemical isoforms of the TR probe which have different mobilities;^24^ however, it is unclear why these two subpopulations would become more apparent in the low-concentration regime. An alternative explanation is that the estimation of TR concentration is less accurate in the low-concentration range, either because of the lower experimental signal-to-noise ratio or due to limitations in the theory used to convert raw fluorescence intensity to concentration. Nevertheless, we emphasize that the fit is good across most of the concentration range, from ∼0.5–7% TR. Overall, our finding that the fit is relatively good suggests that the molecular concentration profile of TR-DHPE does indeed approximate to an exponential growth function if self-quenching effects are accounted for.

## Conclusions

This study showed that electrophoresis can be used to control the organization of charged fluorophores, held within a model membrane via covalent linkages to lipids, and that this can be effectively analyzed via FLIM. A concentration-induced self-quenching of the fluorescent probes was directly observed as the correlation of the accumulation of fluorophores to a reduction in their fluorescence lifetime. Prior to this investigation, researchers have typically studied concentration-related quenching (or energy transfer) by varying the concentration of fluorophores across series of samples.^47−49^ The experimental platform demonstrated here is remarkable for allowing the generation of a continuum of concentrations in a single sample and for showing it is possible to directly visualize the dynamic development of a quenching system, in real-time. By varying the initial concentration of TR fluorophores incorporated into SLBs from 0.3% to 0.8% (mol/mol), we demonstrated the ability to modulate the maximum concentration of fluorophores reached during electrophoresis to between 2% and 7% (mol/mol) which led to a maximal quenching of ∼65%. Other studies have achieved up to 25-fold increases in the concentration of lipid-tethered fluorophores by using different membrane geometries or AC currents,^8,31^ and it seems likely that changing the dimensions of the patterned template or the electrophoresis parameters will allow higher fluorophore concentrations to be achieved. Finally, we demonstrated a method for converting fluorescence intensity profiles into concentration profiles by correcting for quenching effects. This revealed some evidence of subtle lipid–lipid interactions at high packing densities. Overall, these findings show that the combination of in-membrane electrophoresis and FLIM can be effective to assess molecular interactions via the photophysical state of a fluorescent probe. In the future, it would be interesting to compare between different fluorophores to assess how the chemical and photophysical properties of a chromophore lead to different quenching behaviors.^50−52^ Furthermore, it should be possible to test theoretical predictions about how quenching occurs, e.g., the transfer-to-trap model.^15^ Finally, this platform opens the tantalizing possibility of the quantitative analysis of membrane proteins involved in photosynthesis that have critical functions related to excited state energy transfers, such as light-harvesting complexes.^38,39,45,46^

## Data Availability

All relevant raw and analyzed data associated with this paper are openly available under a CC-BY license in the Research Data Leeds repository^53^ and can be found at https://doi.org/10.5518/1284.

## Supporting Information Available

Additional experimental data and associated explanations: (i) fluorescence recovery after photobleaching measurements to confirm membrane quality, (ii) fluorescence images showing that minimal accidental photobleaching occurs, (iii) FLIM analysis of singlet–singlet annihilation effects, (iv) tabulated data of fluorescence-concentration relationship of standard samples, and (v) fluorescence images showing examples of defects in lipid membranes generated during electrophoresis; derivation of equation for converting raw fluorescence intensity to concentration (PDF)

## Abbreviations

DiynePC: 1,2-bis(10,12-tricosadiynoyl)-*sn*-glycero-3-phosphocholine
DOPC: 1,2-dioleoyl-*sn*-glycero-3-phosphocholine
FLIM: fluorescence lifetime imaging microscopy
FRET: Förster resonance energy transfer
TR: Texas Red
TR-DHPE: Texas Red 1,2-dihexadecanoyl-*sn*-glycero-3-phosphoethanolamine
ROI: region-of-interest
SLB(s): supported lipid bilayer(s)
