# Supplementary material for: Self-Quenching Behavior of a Fluorescent Probe Incorporated within Lipid Membranes Explored Using Electrophoresis and Fluorescence Lifetime Imaging Microscopy
Source: J Phys Chem B. 2023 Feb 21;127(8):1715–27. doi: 10.1021/acs.jpcb.2c07652 (PMC9986866; doi:10.1021/acs.jpcb.2c07652)
Supplement: Supplementary file 1 — jp2c07652_si_001.pdf [file jp2c07652_si_001.pdf]

Supporting Information

for the article:

Self-quenching Behaviour of a Fluorescent Probe Incorporated within Lipid Membranes Explored using Electrophoresis and Fluorescence Lifetime Imaging Microscopy

Sophie A. Meredith,[†,‡] *Yuka Kusunoki,*[§] *Simon D. Connell,* [†,‡] *Kenichi Morigaki,* [§]

Stephen D. Evans, [†,‡] *Peter G. Adams**[†,‡]

[†] School of Physics and Astronomy, University of Leeds, Leeds, LS2 9JT, UK

[‡] Astbury Centre for Structural Molecular Biology, University of Leeds, Leeds, LS2 9JT, UK

[§] Graduate School of Agricultural Science and Biosignal Research Center, Kobe University

Rokkodaicho 1-1, Nada, Kobe, 657-8501, Japan

SI 1. Confirming the membrane quality: structural contiguity and lateral lipid diffusion

FLIM images of a DOPC supported lipid membrane containing 0.28 % TR-DHPE (mol/mol relative to DOPC) are shown in **Figure S1A**. Fluorescence is restricted to the well-defined square-patterned corral region, with minimal fluorescence signal located on the surrounding template. The fluorescence within each membrane is largely homogeneous across the corral (40-50 counts/pix for all fluorophores), with a few visible bright spots that may represent non-ruptured vesicles that are loosely adsorbed onto the membrane. Overall, these membranes were highly reproducible, with minimal variation in intensity and quality across multiple preparations. It was important to confirm that these membranes were of high quality, i.e., a contiguous lipid bilayer that allows microscale lateral diffusion of lipids, To do this, fluorescence recovery after photobleaching (FRAP) experiments were performed to monitor the diffusion of lipids over time. For each sample, a circular area (with a bleached radius, R_{bleach}, ranging from 20 – 30 µm) of membrane was deliberately photobleached using intense white light. Immediately after photobleaching, a FLIM timelapse of images (**Figure S1B**) was obtained to monitor the diffusion of "non-bleached" fluorophores into the bleached area. The intensity of the fluorescence recovery in the bleached spot was monitored for each timepoint (each time point is the accumulation of photons in a 16 s period) to plot a fluorescence recovery curve (**Figure S1C**). A mono-exponential fit, $F = F_0(1-e^{-kt})$, was used to obtain the "doubling time", $\tau = ln(2)/k$, for each sample, from which the diffusion constant, $D = 0.22 \times R_{bleach}^2/\tau$, was calculated. To calculate the mobile fraction, images of the corral before photobleaching and after photobleaching were analysed. Briefly, the intensity of the "bleached" region was compared to the intensity of a "non-bleached" region throughout the FRAP experiment. A mobile fraction of 100% would be represented by the two regions having an equal intensity after the system has reached equilibrium. A summary of FRAP experiments on patterned bilayers is shown in **Table S1.** For each fluorophore, the diffusion of the lipids was compared in both patterned bilayers (lipid bilayers ruptured into a 100 µm square template) and in "infinite" bilayers (lipid bilayers ruptured onto non-patterned glass) to ensure that the polymerised lipid template did not adversely affect the lipid mobility. The calculated diffusion constant was similar for TR-DHPE in both patterned and non-patterned SLBs at ~2 $\mu m^2/s$ and the mobile fraction was high (>95%). Overall, these results are consistent with the values for the diffusion constant and mobile fractions of similar lipid-tagged fluorophores reported in other studies [1, 2]. This is an indication that there are only transient interactions between lipids and the glass substrate or between lipids and the diynePC template that do not significantly restrict the 2-D diffusion of the lipids and fluorophores. In addition, the mobility, $\mu_{FRAP} = D/k_BT$, was calculated for each fluorophore (ranging between 4.2 to 5.5 $ms^{-1}N^{-1}$). The mobility is a measure of how a particle responds to an applied force.

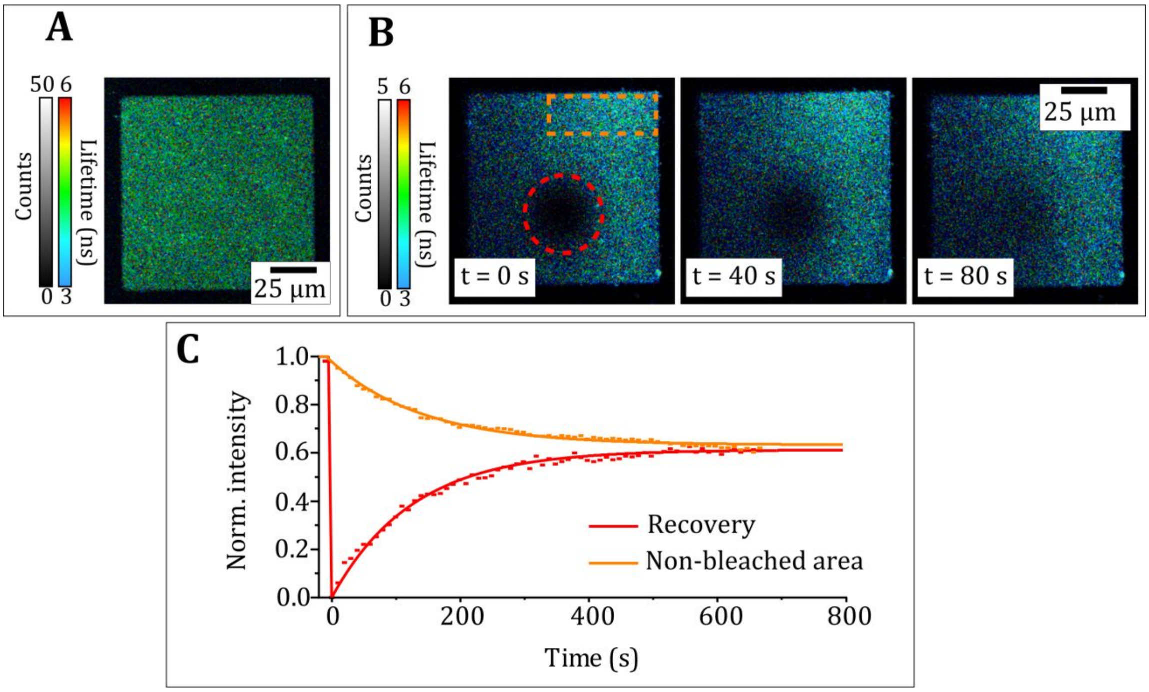

Figure S1: *FLIM images of TR -containing lipid bilayers and Fluorescence Recovery After Photobleaching (FRAP) experiments confirming that these fluorophores are mobile and a suitable target for electrophoresis.* ***(A)*** *Example image of DOPC SLBs containing 0.28 % (mol/mol) of TR formed as 100 × 100 µm corrals within the DiynePC polymer templates.* ***(B)*** *FRAP experiment for the patterned SLBs, showing the mobility of each type of fluorophore after photo-bleaching.* ***(C)*** *FRAP recovery curves showing the fluorescence intensity in the dashed bleached region in (b), normalised to the intensity of the bilayer prior to photobleaching (colours as labelled on legend). The orange curve shows the fluorescence intensity decrease in the orange, box region in (b), showing that the intensity in the patterned membrane decreases due to there being a finite number of non-bleached fluorophores available to diffuse into the bleached region.*

Parameter	Patterned bilayer	Infinite bilayer
D ($\mu m^2 s^{-1}$)	2.27 ± 0.59	1.95 ± 0.20
μ_{FRAP} ($ms^{-1}N^{-1}$)	5.49 ± 0.14	4.71 ± 0.48
Mobile %	95.5 ± 2.50	96.6 ± 3.60

Table S1: *Mobility data from FRAP experiments, comparing patterned lipid bilayers (100 µm corrals) versus "infinite" bilayers (not confined, i.e., simply assembled on piranha-cleaned glass coverslips without any template). Overall, the prescence of the DiynePC template has no significant effect on the mobility of the TR lipids.*

SI 2. Optimization of FLIM acquisition parameters to minimize photobleaching

Photobleaching can cause the irreversible damage to fluorophores which has the consequence of altering the concentration of "active fluorophores" in an undesirable manner. Therefore, it is important to limit the effects of photobleaching to avoid misinterpretation of fluorescence microscopy data. To quantify the average amount of photobleaching that occurs during the acquisition of a standard FLIM image (25 frames, 80 s of exposure), membrane corrals containing TR were imaged via FLIM for a prolonged acquisition (250 frames) and then digitally separated into images consisting of 25 frames each. Example FLIM images are shown for a membrane corral containing 0.28% (mol/mol) TR at increasing timepoints in a photobleaching experiment (**Figure S2A**), in which the fluorescence signal remains approximately constant throughout. The intensity (normalised at t = 0) per image was plotted for each fluorophore (**Figure S2B**) and shows that the fluorescence intensity for TR decreases to 91.7 % of its initial intensity after a 250 frame acquisition. By fitting an mono-exponential decay curve, $A = A_0e^{-kt}$, to each data set, the bleaching rate, k, for the TR fluorophore was found to be 0.133×10^{-3} s^{-1} for TR and the average amount of bleaching in a standard 25-frame acquisition was 1.16 %. For subsequent FLIM analysis reported in this manuscript we acquired 25 frames (80 s of exposure). Overall, these results show that the amount of photobleaching that occurs during FLIM measurements with these optimized acquisition parameters is low, and we can be confident that collecting FLIM data in this manner does not significantly affect the concentration of "active fluorophores".

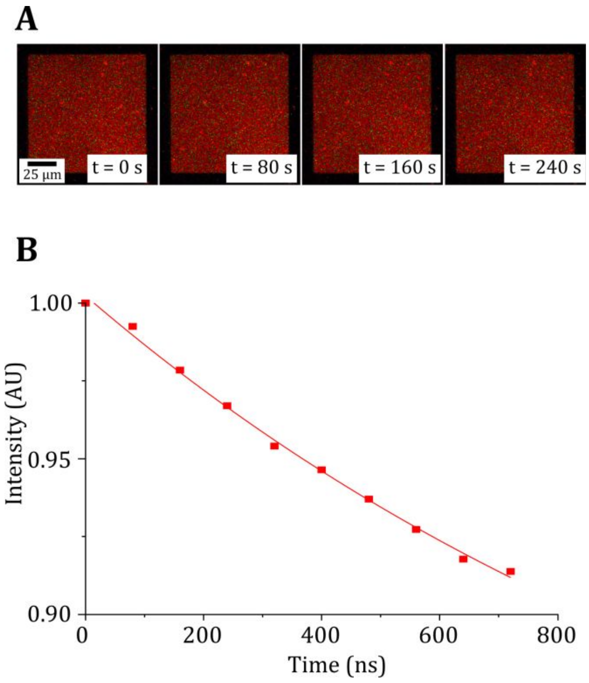

Figure S2: *Photobleaching curves showing photobleaching of TR fluorescence during FLIM acquisitions relevant for in-membrane electrophoresis (same parameters, except over and extended duration).* ***(A)*** *Example FLIM images of a patterned bilayer containing 0.28 % (w/w) TR during a photobleaching experiment.* ***(B)*** *Normalised intensity versus time plots from photobleaching experiments for TR. The photobleaching rate, k, was found from fitting a mono-exponential decay (solid lines),* $A = A_0e^{-kt}$*, to the data set.*

SI 3. Optimization of FLIM acquisition parameters to avoid exciton annihilation effects

When two excitons "meet" each other (after energy transfer) they can annihilate. Singlet-singlet exciton annihilation effects can distort fluorescence measurements and reduce the fluorescence lifetime values measured. The probability of annihilation increases with laser power, therefore, the most straightforward method to avoid significant annihilation effects is simply to decrease laser power. However, this comes at the expense of fluorescence signal (intensity). Therefore, there must be a compromise. For our FLIM instrument, using a 561 nm laser for excitation, with a 10 MHz repetition rate and a pulse width of ~70 ps, we typically have a laser spot diameter of ~800 nm. Excitation fluences (power per unit area during the pulse) can be calculated from this.

Figure S3A shows that images of SLBs undergoing electrophoresis were very similar at all excitation fluences from 0.001 - 0.117 mJ cm^{-2}. The fluorescence intensity and lifetime were broadly similar up to 0.011 mJ cm^{-2} (1-5% lower). An order of magnitude highly fluence produced significant reduction in fluorescence intensity (25%) and fluorescence lifetime (2.5 ns vs. 2.8 ns at the left-edge of the corral). On balance, it was decided that an excitation fluence of 0.012 mJ cm^{-2} provided sufficient fluorescence signal to produce good images and appeared to limit any singlet–singlet annihilation events, as shown in **Figure S3**.

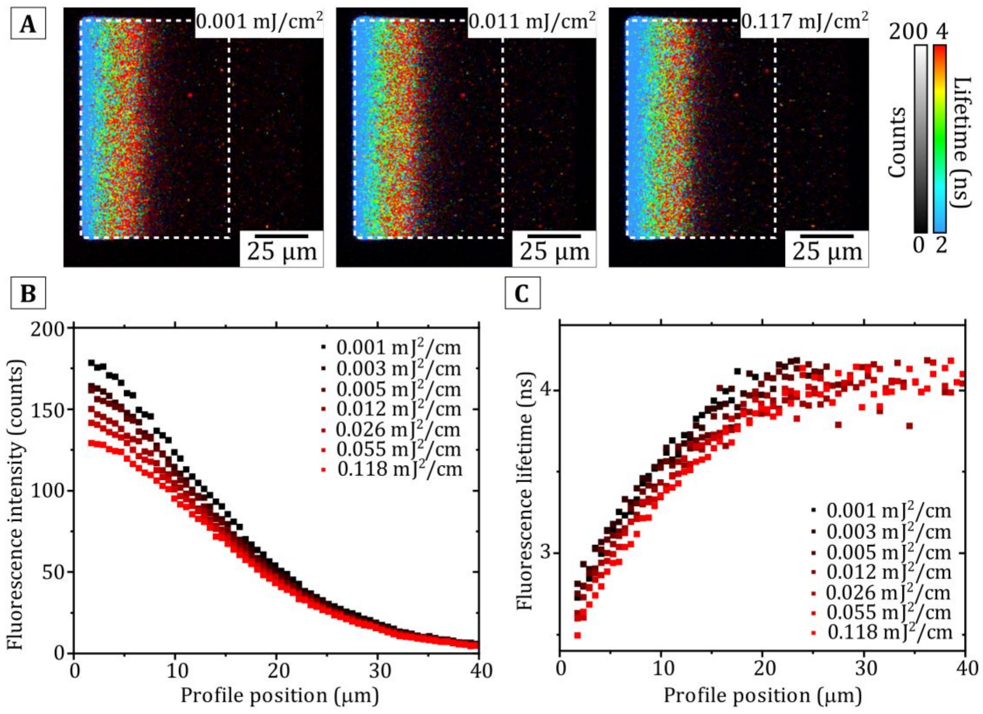

Figure S3: *FLIM analysis revealing the occurrence of subtle singlet-singlet annihilation of excitation for TR fluorophores.* ***(A)*** *Series of FLIM images of corrals of SLBs containing 0.28% (mol/mol) TR during in-membrane electrophoresis, showing the same area of membrane taken at different laser fluences. The white dashed boxes show the area of the image that was used to obtain intensity and lifetime profiles.* ***(B)*** *Intensity profiles obtained from the white dashed regions in (a). Increasing laser fluence is represented by the black to red colour scheme, as annotated.* ***(C)*** *Lifetime profiles obtained from the white dashed regions in (a). Increasing laser fluence is represented by the black to red colour scheme, as annotated.*

SI 4. Theoretical model relating self-quenching of fluorophores to their concentration

To interpret experimental data that involves the self-quenching of fluorescent probes we consider a mathematical model that was previously established and adapt this for our own purposes. Below, is the derivation of an expression which relates the quenched and non-quenched fluorescence intensity to fluorescence lifetime for any given fluorophore (Eq. S12 = main text Eq. 1).

First, we recognize that the density of fluorophores can be expressed either a number density or a mole-to-mole ratio:

$$C_N = \frac{C_{\%}}{100} \times \frac{1}{A_{lipid}} \qquad \text{Eq. S1}$$

where C_N and $C_{\%}$ are the concentration of fluorophores as molecules per nm^2 (i.e., the areal density) and as a % mole-to-mole, respectively, and A_{lipid} is the area occupied by a single lipid (estimated to be 0.67 nm^2).[3]

Next, we note that the average distance between molecules (r) can be related to concentration via the area that it would occupy, from geometry of a circle:

$$\text{Effective area per molecule } = \frac{1}{C_N} = \pi r^2 \qquad \text{Eq. S2}$$

Now that concentrations and distances have been considered, we must apply a theoretical model for concentration-dependent self-quenching. Numerous studies have proposed that self-quenching processes involve a combination of two important processes: excited state transfer and energy dissipation. The overarching idea is that an exciton may migrate between multiple fluorophores and finally become quenched at a "trap site".[4, 5] Transfer-to-trap quenching will affect the observed fluorescence intensity and lifetime in a combination of processes, as follows:

(i) Traps do not fluoresce in response to direct excitation because photon absorption is forbidden, thus, *the overall fluorescence intensity is reduced by the fraction of fluorophores involved in trap sites* but the fluorescence lifetime is unaffected. This is sometimes termed "static quenching".

(ii) Fluorophores that are not part of traps may become excited and then transfer this energy to a trap site, via resonance energy transfer (FRET), whereupon the energy is immediately dissipated (non-radiatively). In this process, the trap provides an alternative route for the rapid dissipation of energy, resulting in *a reduction in both the fluorescence emission intensity and lifetime*. This is termed "transfer-to-trap quenching".

These two processes will occur simultaneously in a system that undergoes concentration-quenching, so that the fluorescence intensity is reduced by (i) and (ii), whereas the lifetime is only affected by (i).

Our theoretical model will consider a random distribution of molecules that represent the situation of traps being "statistical pairs" of fluorophores (aggregation of fluorophores would complicate the situation and is outside the scope of the current study). The probability of trap formation (in the absence of aggregation) is equated to the probability of interactions between randomly distributed particles in a 2-D plane, which can be described using the two-dimensional Perrin equation. In this way, the fraction of fluorophores that are part of traps, f_T, is given by:

$$f_T = 1 - e^{-\pi R_c^2 C} \qquad \text{Eq. S3}$$

where C is the concentration of fluorophores (in molecules/nm^2) and R_C is the distance at which two fluorophores have a 63% ($1 - e^{-1}$) likelihood to form a trap site, also known as the "critical radius for trap formation".[6] Due to the slow lateral and rotational diffusion of fluorophores relative to their fluorescence lifetime these statistical pairs can be considered as quasi-stable and immobile for the duration of an excited state. The probability that energy transfer occurs from an excited molecule to a trap site can then be expressed as:

$$P_{ETT} = P_{FRET} \times f_T \qquad \text{Eq. S4}$$

where P_{FRET} is the probability of energy transfer, as described by Förster theory[7], and f_T is the fraction of fluorophores involved in traps (from Eq. 3). This expression applies the assumption that excited molecules only interact with a single nearest-neighbour for the purposes of FRET, justified by multiple studies which have shown that energy migration is dominated by nearest-neighbour interactions[8, 9]. Transfer-to-trap quenching is dominated by the number of traps in the membrane and at sufficiently high fluorophore concentrations it is reasonable to assume that $P_{ETT} \approx f_T$ (i.e., that $P_{FRET} \approx 1$). Inter-fluorophore energy transfers increase the overall FRET efficiency making this assumption even more reasonable. Using the approximation that $P_{ETT} = f_T$, simplified relationships can be derived that relate a reduction in fluorescence lifetime and intensity to the statistical models for quenching.

Initially, considering that the fluorescence lifetime is only affected by transfer-to-trap quenching, the relative change in lifetime should be equal to:

$$\frac{\tau}{\tau_0} = 1 - P_{ETT} \quad \text{Eq. S5}$$

And if $P_{ETT} = f_T$ and $f_T = 1 - e^{-\pi R_c^2 C}$ (Eq. S3) then:

$$1 - P_{ETT} = e^{-\pi R_c^2 C} \quad \text{Eq. S6}$$

So:

$$\frac{\tau}{\tau_0} = e^{-\pi R_c^2 C} \quad \text{Eq. S7}$$

This can be written in a semi-logarithmic format to provide a linear relationship between fluorophore concentration and the amount of quenching:

$$ln\left(\frac{\tau_0}{\tau}\right) = \pi R_c^2 C \quad \text{Eq. S8}$$

Secondly, considering that the fluorescence intensity is affected by *both* transfer-to-trap quenching *and* static quenching by non-fluorescent dimers, the relative intensity is expected to be equal to:

$$\frac{F}{F_0} = f_T(1 - P_{ETT}) \quad \text{Eq. S9}$$

This equation can also be expressed in terms of the fluorophore concentration by multiplying Eq. S3 with Eq. S6:

$$\frac{F}{F_0} = e^{-2\pi R_c^2 C} \quad \text{Eq. S10}$$

and, again, by rearranging and taking the natural logarithm:

$$ln\left(\frac{F_0}{F}\right) = 2\pi R_c^2 C \quad \text{Eq. S11}$$

Finally, we can derive an equation for the expected "non-quenched" fluorescence intensity (*F_0*) in terms of the raw fluorescence intensity (*F*) and the ratio τ/τ_0 by combining Eq. S8 and Eq. S11 and rearranging for *F_0*:

$$F_0 = F \cdot e^{\left[2\ln\left(\frac{\tau_0}{\tau}\right)\right]} \quad \text{Eq. S12}$$

This equation (Eq. S12) is the equation used in the main text as Eq. 1.

SI 5. Conversion of raw fluorescence intensity data to molecular concentration by using a standard curve and correcting for the effects of quenching

Supported lipid bilayers containing different chromophore concentrations, in a range from 0.1 – 0.8% (mol/mol) TR were formed on hydrophilic glass, as described in the main text. FLIM images are shown in main text **Figure 3A**. These images were analysed by measuring the raw fluorescence intensity and the fluorescence lifetime measured as an average of all pixels in the image. The tabulated data is shown below in **Table S2** and is plotted in **Figure 3B** of the main text.

This analysis makes the reasonable assumption that the ratio of lipid-linked fluorophores to DOPC lipids does not change during sample preparation. In other words, that the ratio of fluorophore-to-lipid in the liposomes is equal to the ratio of lipids that were initially mixed in organic solvent. It does not assume 100% yield of material is retained, because only the *ratio* of fluorophore-to-lipid will affect the number of fluorophores per unit area (areal density). It is likely that we have high accuracy when measuring out quantities of DOPC because we are able to weigh the lipids after drying them into glass vials. It is possible that our error in the *absolute* values for fluorophore concentrations is up to 10% because these are supplied at very low masses (100 µg or 1 mg) which are challenging to validate. However, the *relative* differences between concentrations will be highly accurate because these were prepared from the same stock of each fluorophore.

**concentration (%mol/mol)**	**F (counts/pix)**	**$<\tau>$ (ns)**	**F_0 (counts/pix)**
0.14	31.7	4.23	31.3
0.28	57.5	4.07	61.2
0.42	79.4	4.07	84.6
0.57	93.1	3.93	106.3
0.71	105.0	3.82	126.9
0.85	112.0	3.59	153.3

Table S2: *Calculation of the "non-quenched" fluorescence intensity as a function of fluorophore concentration for TR. The raw fluorescence intensity, F, and the fluorescence lifetime, τ, are measured for each image (average of all pixels in one 25-frame FLIM acquisition). The non-quenched intensity, F_0, is calculated from F and τ using the following equation (Eq. S12):* $F_0 = F \cdot e^{\left[2\ln\left(\frac{\tau_0}{\tau}\right)\right]}$. τ_0 *was set as equal to 4.2 ns, the lifetime value from the lowest concentration measured, where quenching was shown to be negligible.*

SI 6. Lipid membrane disruption and possible lipid tubule formation at high TR concentrations

As highlighted in main text **Figure 5B** (*white arrow*), lipid bilayer disruption sometimes occurred during electrophoresis of corrals containing 1.5 % TR. This bilayer disruption occurred for the majority (80%, N = 10) of membranes containing 1.5 % TR after electrophoresis. Multiple examples are shown in **Figure S4A**, below. This disruption typically resulted in highly mobile strand-like features that stem from microscale regions lacking fluorescence (~5-20 µm in diameter) within the membrane. These were thought to be lipid tubules because of their similarity in appearance with previous reports[10, 11], i.e., tubes of lipid bilayer 50-100 nm diameter and many microns in length. TR-DHPE is a charged lipid with a large headgroup relative to the hydrophobic portion of the lipid (see structure in main text **Figure 1B**). It is therefore likely that high concentrations of TR induce membrane curvature and defects due to imperfect packing of a mixture of TR-DHPE and DOPC lipids[12], or due to electrostatic repulsion between fluorophores that are not sufficiently screened[10, 13]. At sufficiently high concentrations of TR-DHPE, this may result in instability of the lipid bilayer, delamination of the bilayer from the underlying glass substrate, or even extreme curvature of the bilayer and lipid tubulation (as illustrated in **Figure S4B**). It seems feasible that these weakened areas of membrane may be washed away from the substrate by the shear force due to the constant flow of the buffer parallel to the membrane (this buffer flow is required for electrophoresis to prevent the build-up of ions and bubbles at the electrodes), resulting in microscale holes in the membrane (see *right-hand panel* in **Figure S4A**). The membrane defects are consistently observed to be positioned roughly in the centre of the corralled bilayer. This suggests that the polymer template may help to stabilise lipid membranes, and that it may be possible to design templates with different dimensions to better support highly curved lipid bilayers and achieve even higher concentrations of charged fluorophores. In the present study (using 100 × 100 µm square-patterned SLBs), these membrane defects show that experimental limit for maximum concentration of fluorophores in a (quasi-)stable lipid bilayer structure has been reached.

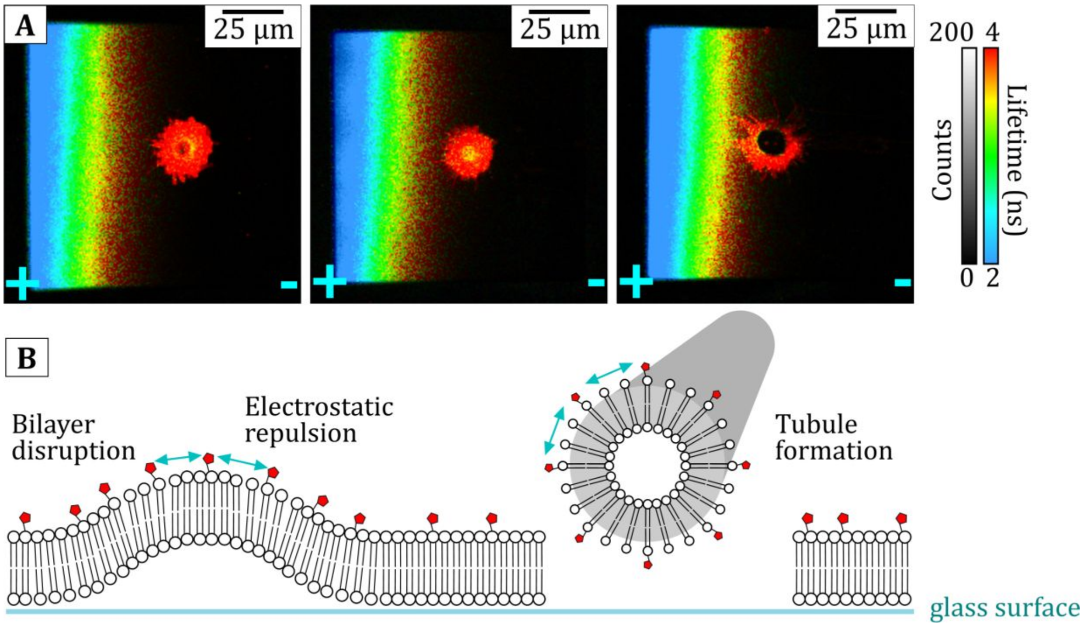

Figure S4: *Examples images from different corrals on the same sample of membrane disruption resulting from in-membrane electrophoresis of TR in lipid membranes.* ***(A)*** *FLIM images of membrane corrals containing 0.84 mol/mol% TR-DHPE to DOPC, in equilibrium in a 45 V/cm field, showing examples of membrane disruption.* ***(B)*** *Schematic showing possible mechanisms for membrane disruption by a high concentration of charged fluorophores. Charged fluorophores may repel each other (blue double-ended arrows) causing membrane curvature and a delamination of the bilayer from the solid support. At sufficiently high membrane curvatures, the lipid bilayer may form tubules that extend from the surface by many micrometres.*

SI 7. Detailed analysis of TR fluorescence decay curves and their exponential fits

Fluorescence decay curves represent the excited state decay of the population of fluorophores. In this study, these curves reveal how the population of Texas Red molecules are interacting. A mono-exponential decay function would be expected for if all TR undergo decay by fluorescence, whereas the appearance of additional quenching pathways would be expected to lead to a multi-exponential decay. FLIM images can be analysed in the SymPhoTime software using one of two processes: (1) the lifetime can be calculated for all pixels in the images to provide a "lifetime image" by fitting the data in each pixel to a multi-exponential decay function or, (2) a region-of-interest (ROI) can be selected and a single decay curve generated to represent this ROI and fit to a multi-exponential decay function. Process 1 was used in the majority of this study because a pixel-by-pixel lifetime is required to analyse spatial distributions of lifetimes, i.e., how the TR changes with distance across an image. Decay curves in each pixel are relatively noisy and were not exported from the analysis software and the accuracy comes from averaging many pixels (the vertical-averaging explained in main text Figure 3). Process 2 accumulates the data from many pixels but it produces decay curves that have higher signal and are preferable to assess the (multi-)exponential character of the decay. As noted, this multi-exponential character can reveal quenched states. Therefore, to assess how the excited states of TR change and explore how quenched states occur at different TR concentrations, we analysed the FLIM data in ROIs in membrane corrals before and after electrophoresis (**Figure S5A-B**).

A cursory observation of the fluorescence decay curves for the initial state of membrane corrals before any electrophoresis (**Figure S5C**) shows a slow monoexponential decay at the lowest TR concentration (0.28%) and a slightly more rapid decay at higher TR concentrations (0.57 and 0.85%). In contrast, the decay curves from ROI at the left-edge of the corral after electrophoresis (**Figure S5D**) reveal a much more rapid decrease and appear to be biexponential, rather than monoexponential. This is particularly clear for the samples with higher initial TR concentrations. A deeper look at the exponential fit parameters reveals a trend for two lifetime components where the relative amplitudes vary with changes to the TR concentration. Before electrophoresis, the membrane at 0.28% TR has a monoexponential decay with a lifetime of ~4.0 ns whereas at 0.57%/0.85% TR there is the appearance of a second decay component at 1.9-2.0 ns (**Figure S5E**, $\tau 2$) that has an amplitude of 18-21% of the overall decay (**Figure S5G**, *A2*). This suggests that at high TR concentrations in these membrane corrals, there is already a small amount of TR quenching due to TR-TR interactions, even before electrophoresis. This could be physical aggregates of TR or simply a reduction in TR-TR distance leading to FRET and energy-dissipation at traps due to "statistical pairs" of TR.[4-6] After electrophoresis the best fit is produced by fitting biexponential functions, and the lifetime of the long component decreases from 3.6 to 2.8 ns (**Figure S5F**, $\tau 1$) and the short component decreases from 2.0 to ~1.2 ns (**Figure S5F**, $\tau 2$) with increasing TR concentrations. The amplitude of the short component increases from 20% to 52% to 63% (**Figure S5H**, *A2*) as the starting TR concentration increased from 0.28% to 0.57% to 0.85%. The full fit parameters are shown in **Table S3**. Overall, this suggests that the energetic state of the TR changed as it accumulated and that the biexponential function could represent either two distinct populations of TR states (e.g., non-quenched and quenchers) or a continuum of TR states (e.g., quenchers connected to a greater or lesser extent to non-quenched TR) that just happen to produce a good fit to a biexponential function.

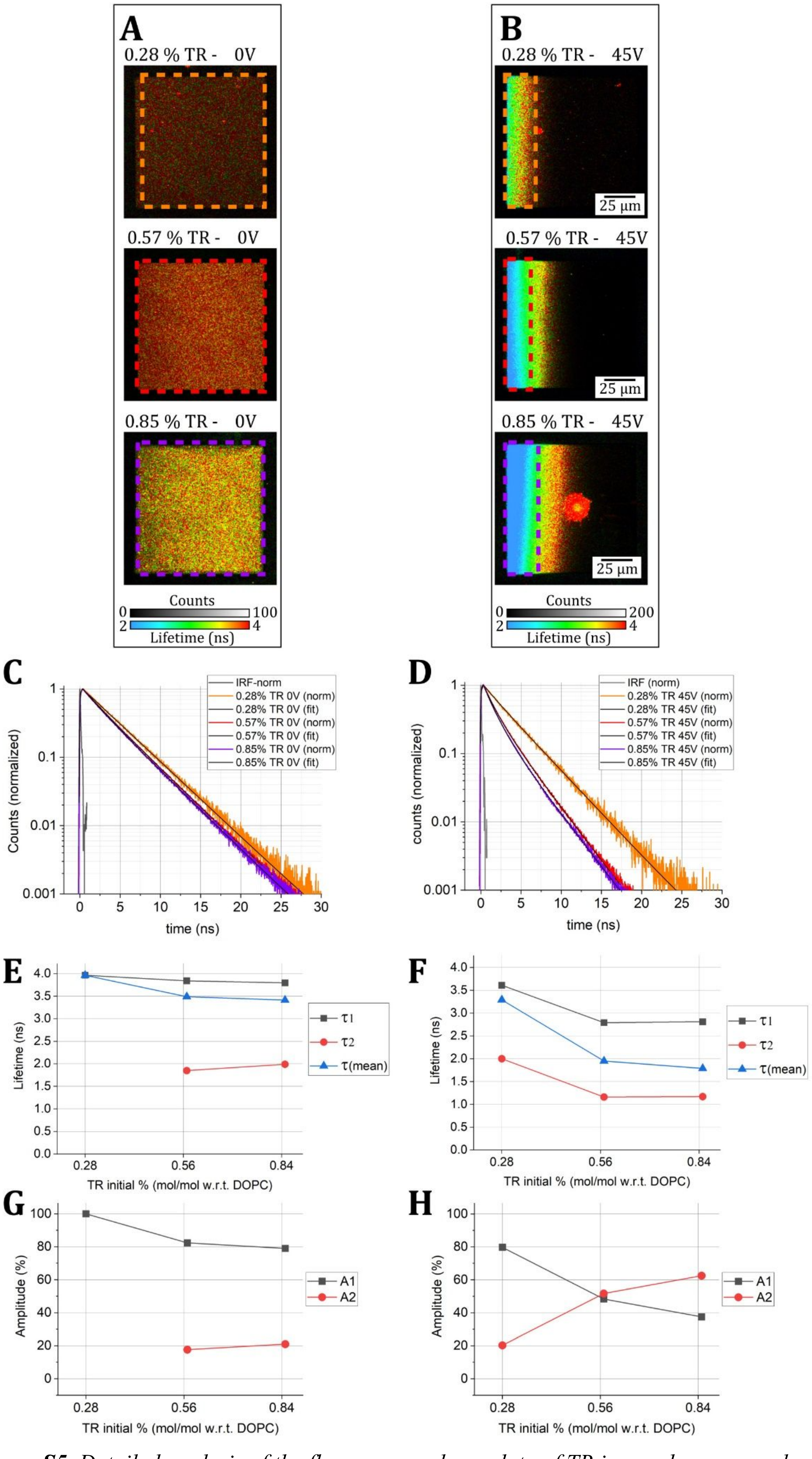

Figure S5. *Detailed analysis of the fluorescence decay data of TR in membrane corrals.*

Figure S5 (caption continued). *FLIM images of membrane corrals either before* ***(A)*** *or after* ***(B)*** *electrophoresis. The starting TR concentration is noted. Panels (A)-(B) are reproduced from* ***main text Figure 5*** *and annotated to show the ROI selected for further analysis. Fluorescence decay curves* ***(C)-(D)*** *from the ROIs shown in (A)-(B). Graphs* ***(E)-(F)*** *plotting how the lifetime value of changes with TR% for the short component, long component or the mean of the decay fits from (C)-(D). Graphs* ***(G)-(H)*** *plotting how the percentage amplitude changes with TR% for the short or long lifetime component. The graphs were plotted using the data tabulated in* ***Table S3***.

Sample	A1 [%]	τ1 [ns]	A2 [%]	τ2 [ns]	<τ>* [ns]	X^2
0.28% TR 0V	100.0	3.97			3.97	1.01
0.28% TR 45V	79.7	3.61	20.3	2.00	3.29	1.01
0.57% TR 0V	82.4	3.84	17.7	1.85	3.49	1.16
0.57% TR 45V	48.3	2.79	51.7	1.16	1.95	1.25
0.85% TR 0V	79.0	3.80	21.0	1.99	3.41	1.27
0.85% TR 45V	37.5	2.81	62.5	1.17	1.79	1.26

Table S3. *Fit parameters from the analysis of the regions-of-interest defined in Figure S5.*
Sample: the initial TR mole/mole ratio is noted as a percentage and 0V or 45V represents before or after (respectively) the application of a 45V/cm E-field.
A1 and A2: the amplitudes of the lifetime components τ1 and τ2.
X^2 (chi-squared): this value represents the quality-of-fit and <1.3 is typically considered reasonable.
**amplitude-weighted mean lifetime.*

Supporting Information Reference List

(1) van Weerd, J.; Krabbenborg, S. O.; Eijkel, J.; Karperien, M.; Huskens, J.; Jonkheijm, P., On-chip electrophoresis in supported lipid bilayer membranes achieved using low potentials. *J. Am. Chem. Soc.* **2014,** *136*, 100-103.

(2) Bao, P.; Cheetham, M. R.; Roth, J. S.; Blakeston, A. C.; Bushby, R. J.; Evans, S. D., On-chip alternating current electrophoresis in supported lipid bilayer membranes. *Anal. Chem.* **2012,** *84*, 10702-10707.

(3) Hills, R. D.; McGlinchey, N., Model parameters for simulation of physiological lipids. *J. Comput. Chem.* **2016,** *37*, 1112-1118.

(4) Boulu, L. G.; Patterson, L. K.; Chauvet, J. P.; Kozak, J. J., Theoretical investigation of fluorescence concentration quenching in two-dimensional disordered systems. Application to chlorophyll a in monolayers of dioleylphosphatidylcholine. *J. Chem. Phys.* **1987,** *86*, 503-507.

(5) Brown, R. S.; Brennan, J. D.; Krull, U. J., Self-quenching of nitrobenzoxadiazole labeled phospholipids in lipid membranes. *J. Chem. Phys.* **1994,** *100*, 6019-6027.

(6) Chandrasekhar, S., Stochastic problems in physics and astronomy. *Rev. Mod. Phys.* **1943,** *15*, 1-89.

(7) Forster, T., Delocalization excitation and excitation transfer. *Mod. Quantum Chem.* **1965**.

(8) Baumann, J.; Fayer, M. D., Excitation transfer in disordered two-dimensional and anisotropic three-dimensional systems: Effects of spatial geometry on time-resolved observables. *J. Chem. Phys.* **1986,** *85*, 4087-4107.

(9) Knoester, J.; Van Himbergen, J. E., Monte Carlo simulations on concentration self-quenching by statistical traps. *J. Chem. Phys.* **1987,** *86*, 3577-3582.

(10) Adams, P. G.; Lamoureux, L.; Swingle, K. L.; Mukundan, H.; Montano, G. A., Lipopolysaccharide-induced dynamic lipid membrane reorganization: Tubules, perforations, and stacks. *Biophys. J.* **2014,** *106*, 2395-2407.

(11) Farsad, K.; De Camilli, P., Mechanisms of membrane deformation. *Curr. Opin. Cell Biol.* **2003,** *15*, 372-381.

(12) Skaug, M. J.; Longo, M. L.; Faller, R., The impact of Texas Red on lipid bilayer properties. *The J. Phys. Chem. B* **2011,** *115*, 8500-8505.

(13) Adams, P. G.; Swingle, K. L.; Paxton, W. F.; Nogan, J. J.; Stromberg, L. R.; Firestone, M. A.; Mukundan, H.; Montano, G. A., Exploiting lipopolysaccharide-induced deformation of lipid bilayers to modify membrane composition and generate two-dimensional geometric membrane array patterns. *Sci. Rep.* **2015,** *5*, 10331.
